# Roles for E1-independent replication and E6-mediated p53 degradation during low-risk and high-risk human papillomavirus genome maintenance

**DOI:** 10.1371/journal.ppat.1007755

**Published:** 2019-05-13

**Authors:** Isao Murakami, Nagayasu Egawa, Heather Griffin, Wen Yin, Christian Kranjec, Tomomi Nakahara, Tohru Kiyono, John Doorbar

**Affiliations:** 1 Department of Pathology, University of Cambridge, Tennis Court Road, Cambridge, United Kingdom; 2 Department of Obstetrics and Gynecology, Keio University School of Medicine, Shinjuku-ku, Tokyo, Japan; 3 Division of Carcinogenesis and Cancer Prevention, National Cancer Center Research Institute, Tokyo, Japan; Fred Hutchinson Cancer Research Center, UNITED STATES

## Abstract

Human papillomaviruses (HPV) have genotype-specific disease associations, with high-risk alpha types causing at least 5% of all human cancers. Despite these conspicuous differences, our data show that high- and low- risk HPV types use similar approaches for genome maintenance and persistence. During the maintenance phase, viral episomes and the host cell genome are replicated synchronously, and for both the high- and low-risk HPV types, the E1 viral helicase is non-essential. During virus genome amplification, replication switches from an E1-independent to an E1-dependent mode, which can uncouple viral DNA replication from that of the host cell. It appears that the viral E2 protein, but not E6 and E7, is required for the synchronous maintenance-replication of both the high and the low-risk HPV types. Interestingly, the ability of the high-risk E6 protein to mediate the proteosomal degradation of p53 and to inhibit keratinocyte differentiation, was also seen with low-risk HPV E6, but in this case was regulated by cell density and the level of viral gene expression. This allows low-risk E6 to support genome amplification, while limiting the extent of E6-mediated cell proliferation during synchronous genome maintenance. Both high and low-risk E7s could facilitate cell cycle re-entry in differentiating cells and support E1-dependent replication. Despite the well-established differences in the viral pathogenesis and cancer risk, it appears that low- and high-risk HPV types use fundamentally similar molecular strategies to maintain their genomes, albeit with important differences in their regulatory control. Our results provide new insights into the regulation of high and low-risk HPV genome replication and persistence in the epithelial basal and parabasal cells layers. Understanding the minimum requirement for viral genome persistence will facilitate the development of therapeutic strategies for clearance.

## Author summary

Human papillomavirus research has been prioritised towards understanding the progression of high-risk HPV infections to malignant cancer, rather than the regulation of the HPV productive cycle. Low-risk HPV types are often considered as a ‘less effective’ version of their high-risk counterparts. To date, they have been inadequately investigated and have generally served as reference viruses that lack oncogenic HPV functions. Here we carried out a comparative analysis using the most prominent high-risk (HPV16) and low-risk (HPV11) HPV papillomaviruses, focusing on viral genome replication and maintenance in the epithelial basal layer. During the maintenance phase, the E1 viral helicase of both HPV types are dispensable. Neither E6 nor E7 proteins are required for the maintenance replication. Once cells become confluent, replication switches from an E1-independent to an E1-dependent mode and requires p53 degradation by E6. Interestingly, the characteristic ability of high-risk E6 to degrade p53 and inhibit keratinocyte differentiation was also seen with low-risk HPV E6, but in this case was regulated by cell density and the extent of viral gene expression. Despite the differences in the pathogenicity of low- and high-risk HPV types, they appear to follow similar molecular strategies to complete viral life cycle.

## Introduction

Papillomaviruses (PV) are small double-stranded DNA viruses which infect stratified epithelia, with the completion of their life cycle being intimately linked to the terminal differentiation program of keratinocytes. One of the most distinctive characteristics of papillomaviruses is their genotype-specific host-restriction, and the preference shown by different papillomavirus types for distinct anatomical sites[1–3]. Human papillomaviruses (HPVs), which consist of more than 200 HPV types, are subdivided into 5 genera, and within the alpha genus, HPVs are further divided into low- and high-risk types based on the cancer risk associated with their infection [4, 5].

HPVs express a relatively small number of early and late viral genes during their life cycle, which are tightly regulated according to the differentiation stage of the infected keratinocytes. The viral genes may be categorized as ‘core genes’ or ‘accessory genes’. All known papillomaviruses have evolved a group of core genes that were present early during papillomavirus speciation, which share similarities in sequence and protein function. The core genes play primary roles in viral genome replication and amplification (E1 and E2) as well as virion assembly (L1 and L2) [6–10]. In contrast, the accessory genes (E6, E7 and E5), which are often referred to as viral oncogenes, have evolved in each papillomavirus type as it adapted to different epithelial niches [11–13]. The sequence and function of these genes are similar yet divergent between types compared to the core genes. In general, accessory genes are involved in modifying the cellular environment in order to complete the viral life cycle at the site of infection. Accessory genes play a variety of important functions at different stages of the viral life cycle, such as the maintenance of infection in the basal layer and the amplification of viral genomes in the suprabasal layers. As such, the function of these accessory genes as well as their regulation may largely attribute to virus diversity in tropism and pathogenicity [5, 6]. In fact, the deregulation of the accessory genes of high-risk HPV can mediate the progression to invasive cancer [4, 14].

The high-risk HPV-mediated carcinogenic process has drawn most of the research effort in the past decades, leaving open important questions about the comparative analysis of the evolutionary adaptation of different HPV types in relation to their specific epithelial sites of infection. Among the best characterized functions of high-risk HPVs there are the E6-mediated degradation of p53 and PDZ domain-containing proteins and the activation of hTERT (reviewed in [12]). High-risk HPV E7 is known to interact and destabilise pRB family proteins, promoting cell cycle re-entry in the parabasal layers of the epithelium and above (reviewed in [11]). Furthermore, both E6 and E7 expressed by high-risk HPV types have multiple additional functions, such as the inhibition of differentiation, apoptosis and host-immune response. In many cases, low-risk HPV E6 and E7 are considered as a ‘de-potentiated’ version of their high-risk counterparts. These HPV types are maintained and propagated in the general population without having the carcinogenic properties of high-risk HPVs, indicating that the induction of cancer is a collateral effect of the life cycle strategy of high-risk HPVs rather than an adaptation.

In this study, we have performed a comparative analysis of how ‘core’ and ‘accessory’ proteins cooperate in promoting the replication of both low- and high-risk viral episomes. We have used HPV11 and 16 as the key prototypes of low- and high-risk alpha HPV types respectively, and used the immortal and isogenic NIKS keratinocyte cell line as a cell model to draw direct comparisons of the life cycle strategies used by the two viruses. We have identified a two-phase HPV replication mode common to both low- and high-risk alpha papillomaviruses that is regulated by cell density. Our data shows that viral replication switches from requiring E2 and being E1-independent while genomes are maintained, to being E1-dependent as keratinocytes commit to differentiation at high cell density, a situation that also requires the function of E6 and E7. Curiously, the low-risk E6 protein was necessary in order to sustain low-risk HPV genome copy number as cell confluence was reached, and that like high-risk E6, this was dependent on E6-mediated p53 proteasomal degradation. Our results suggest that for both high and low-risk HPVs, that E1 is non-essential for synchronous HPV genome replication during maintenance, and that the low-risk E6 protein has a p53-degradation function that is regulated at cell confluence, and which like that high-risk HPV types, can inhibit commitment to differentiation and stimulate cell cycle entry.

## Materials and methods

### Cell culture

293T (ATCC) were maintained in Dulbecco’s Modified Eagle’s Medium (DMEM, SIGMA) supplemented with 10% fetal calf serum (FCS, HyClone) and 1% penicillin and streptomycin. NIKS (a gift from Paul Lambert, McArdle Laboratory for Cancer Research, University of Wisconsin), a HPV-negative spontaneously immortalised human keratinocyte cell line, was maintained at sub-confluence on γ-Irradiated J2 3T3 feeder cells (a gift from Paul Lambert) in F medium with all supplements as previously described [15]. Bromodeoxyuridine (BrdU) was used at a final concentration of 30 μg/ml for 18 hours.

### Plasmid construction and site-directed mutagenesis

The pSPW12 plasmid was a kind gift from Prof. Margaret Stanley (University of Cambridge) containing the HPV16 genome. The pBT-1 clone of HPV11 (Hershey) plasmid was a kind gift from Prof. Neil Christensen (The Pennsylvania State University). HPV16E6SAT, HPV16ΔPBM, HPV16 E1 defective mutant were described previously [16, 17]. All mutant genomes were constructed using a KOD -Plus- Mutagenesis Kit (TOYOBO), prior to DNA sequencing to ensure that no additional base changes were present. The mutants that were constructed in this study comprise the HPV16 E1 helicase defective mutant (K383 to A), HPV16 E6 defective mutant (D9 to STOP), HPV16 E7 defective mutant (L13 to STOP), HPV16 E2 defective mutant (G50 to stop), HPV11E1 defective mutant (D46 to STOP and M47 to STOP), HPV11 E1 helicase defective mutant (K384 to A), HPV11 E6 defective mutant (E2 to STOP), HPV11 E7 defective mutant (L15 to STOP) and the HPV11 E2 defective mutant (L79 to STOP). The primer sequences used for mutagenesis are available upon request.

In order to construct HPV11 and HPV16 reporter genomes, two restriction enzyme recognition sites, *Bgl*II and *Xho*I, were generated in pSPW12 (HPV16), pBT-1 (HPV11) and pSELECT–zeo-GFPBsr (InvivoGen) using the KOD -Plus- Mutagenesis system (Fig 2A, primer sequences available upon request). All mutants were sequenced to ensure that no additional base changes were present. The mutated pSPW12*-Bgl*II/*Xho*I, pBT-1-*Bgl*II/*Xho*I and pSELECT–zeo-GFPBsr-*Bgl*II/*Xho*I were excised from the *Bgl*I and *Xho*I sites. These two flagments, the pSPW12- *Bgl*II/*Xho*I plasmid or pBT-1-*Bgl*I-*Xho*I and GFPBsr-*Bgl*I/*Xho*I, were ligated using Ligation high Ver.2 (TOYOBO).

### Generation of NIKS cell lines containing HPV genomes

Plasmids containing HPV16 and HPV11 genomes (wild type or mutant) were digested with *BamHI* to release the whole viral genome. The linearized HPV11 or HPV16 genomes were then re-circularized and purified as described previously, before being co-transfected with a plasmid encoding blastocidin into NIKS cells [18]. 2x10^5^ cells were seeded in each well of a 6-well plate the day before transfection with F-media incomplete (no EGF). The cells were transfected with 1600 ng of re-circularized HPV DNA, and 400 ng of pcDNA6 encoding a blasticidin resistance gene (Invitrogen), using FuGENE HD (Promega). The next day the cells were seeded onto a 75 cm^2^ flask over blastcidin-resistant feeders with F-media incomplete, then NIKS cells were selected with 4 μg/ml blasticidin S with F-media complete (with 10 ng/ml of EGF) for 4 days and cultured extra 2–3 in the absence of Blastcidin and designated passage one (P1). All experiments were carried out in triplicate using NIKS cell lines containing HPV genomes which were generated at least two independent transfections.

### Monitoring HPV genome replication

3.3x10^5^ NIKS cells containing HPV genomes were seeded in to a 25cm^2^ flask with the same number of feeder cells in F-media complete. Cells were collected for analysis at day 1, 2, 3, 4 and 7. All experiments were done using NIKS cells containing > than 10 copy per cell of each HPV genome at passage 2 post-transfection,.

### Vector construction and retroviral infection

The production and infection of recombinant retroviruses were accomplished as previously described [19]. Construction of retrovirus vectors LXSN-HPV16E6, HPV16E6SAT, HPV16E6ΔPDZ, HPV16E7, HPV16E6E7, HPV11E6, HPV11E7, HPVE6E7 were described previously [20]. Retrovirus vectors of LXSN-HPV11E6, HPV11E7, and HPVE6E7 were constructed by cloning ORF of HPV11 E6 and/or E7 into LXSN using Gateway Recombination cloning technology (Thermo Fisher Scientific) following the manufacturer’s instruction (primer sequences available upon request). LXSN-11E6^W133R^ was constructed using KOD -Plus- Mutagenesis Kit (primer sequences available upon request) and sequenced to ensure that no additional base changes was present. The E6AP-specific shRNA constructs pCL-SI-MSCVpuro-H1R-E6APRi4 was described previously [21]. To generate NIKS cells expressing E6 and/or E7, the cells were seeded 1 day before and inoculated with at MOI of 5 in the presence of 4 μg/ml of Polybrene (Santa Cruz) followed by Geneticin (Thermo Fisher Scientific) selection (400 μg/ml) for 4 days.

### siRNA transfection

For the delivery of siRNAs, 3.3x10^5^ of cells were seeded on 25cm^2^ flasks and transfected 12nM of siRNA using HiPerfect Transfection Reagent (Qiagen) at days 0 and 4. Non-targeting siRNA (MISSION siRNA Universal Negative Control (Sigma)) was used as a negative control and ON-TARGET plus Human TP53 (Dharmacon) was used as a siRNA to p53.

### qPCR and RT-qPCR

Total DNA from NIKS for qPCR was purified using a QIAamp DNA Mini Kit (Qiagen), according to the manufacturer's instructions. All samples were digested with *Dpn*I to remove any residual input DNA prior to analysis. The RNA from NIKS for RT-qPCR was purified by using an RNeasy Mini Kit (Qiagen), and cDNA was synthesised with SuperScript III Reverse Transcriptase (Thermo Fisher scientific) using 100 μM random hexamer primers, according to the manufacturer's instructions. The HPV genome and GAPDH were measured by a ViiA 7 Real-Time PCR System (Life Technologies) using Power SYBR Green/ROX master mix (Thermo Fisher scientific) with 15 min denaturation at 95°C, followed by 45 cycles of 95°C for 15s and 60°C for 60s. The PCR primers for qPCR were as follows. The HPV11 forward primer was 5’-ACATTAGATCCGTGGACAGTACAATC-3’ or 5’-TCGTCCAGCCTAGACATTGAG-3’, HPV11 reverse primer was 5’-TTCCTTCTTTGGTGCTTGTTGTAA-3’ or 5’- TCCAATCGTATGCATTTCCA-3’, HPV16 forward primer was 5’-TGTTTCAGGACCCACAGGAGC-3’, HPV16 reverse primer was 5’- CGCAGTAACTGTTGCTTGCAG-3’, GAPDH forward primer was 5’-CCTCCCGCTTCGCTCTCT-3’, GAPDH reverse primer was 5’-CTGGCGACGCAAAAGAAGA-3’. To quantitate HPV DNA levels in HPV genome-containing cell populations and cell lines, HPV copy number per cell was expressed relative to GAPDH copy number. The human genome blast using designed primers and probe for human GAPDH indicated that there are 4 copies, comprising 2 copies of GAPDH and 2 pseudogene copies of GAPDH per NIKS cell. This was confirmed by determination of GAPDH copy number against known NIKS cell number. The copy number of virus genomes per cell was found to vary at passage 2 between experiments, with HPV11 WT and mutant genomes typically present at around 100 copies per cell. The viral genome copy number per cell at passage 2, day 1 was set at 100%, and subsequent changes in copy number were normalized to this. The quantification of viral gene expression was carried out as described previously [18].

### Immunofluorescence

2.4x10^4^ cells were seeded in each well of a 4-well culture slide (Falcon) and cultured for 3 and 7 days. The cells were washed in PBS and fixed in 4% paraformaldehyde (PFA) in PBS for 30 min. at room temperature. The cells were permeabilised in PBS with 0.1% Triton X-100 (Promega) for 30 min., then washed in PBS. The cells were blocked in 10% normal goat serum (Cell Signaling Technology) in PBS for 1 hour. The antibodies used were anti-Keratin10 antibody (dilution 1:200, Thermo Fisher Scientific), anti-p53 (DO-1) antibody (dilution 1:300, Santa Cruz), anti-MCM antibody (dilution 1:100, Abcam) an anti-mouse Alexa 594-conjugated antibody (dilution 1:150, Thermo Fisher Scientific). For p53 and MCM, the signal was amplified using a Tyramide Signal Amplification Kit (Perkin-Elmer), according to the manufacturer's instructions. Finally, the cells were mounted in mounting medium (Agar Scientific) for visualization. The number of cells with positive signal were quantified using ilastik software.

### SDS-PAGE and Western blotting

Proteins were extracted from cells using RIPA buffer and quantified using the BCA protein assay kit (Pierce), before being separated on 4–12% gradient polyacrylamide-SDS-Tris-Tricine denaturing gel (Invitrogen) and transferred onto PVDF membranes (Bio-Rad). After transfer, membranes were blocked for 1 hour at room temperature in 1% milk in PBS-T (PBS, 0.1% tween20). Blots were then incubated overnight at 4°C with the appropriate primary antibody diluted in 1% milk PBS-T. Primary antibodies used were anti-HPV16E6 (2E-3F8, Euromedex), anti-HPV16E7 (NM2, Santa Cruz), anti-p21 (EA10, Abcam), anti-p53 (DO-1, Santa Cruz), anti-GAPDH (Millipore), followed by the appropriate HRP-conjugated secondary antibody (GE Healthcare), and detection using ECL, or ECL plus kits (GE Healthcare) or by the appropriate IRDye 800CW fluorescent secondary antibody (Licor) followed by detection using an Odissey imaging system (Licor).

### Fluorescent activated cell sorting (FACS)

Sub-confluent NIKS were collected and fixed for 10 min. in 4% PFA in PBS at a concentration of 1x10^6^ cells/ml at room temperature. Immediately before sorting, the cells were passed through a 40μm cell strainer (BD Biosciences). The cells were sorted on a Dako Cytomation MoFlo MLS high-speed cell sorter. DNAs were extracted from the sorted cells as described above.

### p53 reporter assay

5x10^4^ cells were seeded in each well of a 12-well and transfected Cignal p53 Pathway Reporter Assay Kit (Qiagen) at the same day, according to the manufacturer's instructions. The cells were cultured for 3 or 7 days and collected. The activities of firefly and *Renilla* luciferases were measured by a FLUOstar Omega Microplate Reader (BMG LABTECH) using Dual-Luciferase Reporter Assay System (Promega), according to the manufacturer's instructions.

### Southern blot analyses

TDIG-labelled probes comprising the entire HPV11 or HPV16 genome were prepared, and Southern blot analyses were carried out using DIG High Prime DNA Labelling and Detection Starter Kit II (Roche) following the protocol provided by the manufacturer. Briefly, digested DNA was separated on a 0.7% agarose gel, soaked in 0.25 M HCl for 15 min, and alkaline transferred onto nylon membranes (Boehringer Mannheim). The membranes were prehybridized in Hybrisol I (Millipore) for 1 h at 42°C. A DIG-labelled probe was applied for hybridization, and the hybridized DNA was visualized using the detection kit.

### Cesium chloride gradient equilibrium centrifugation

DNA was mixed with cesium chloride (CsCl), and the mixture was adjusted to a volume of 4.5 ml, and a the density of 1.753 g/ml (i.e. corresponding to a refractive index of 1.404). The DNA-CsCl solution was transferred to Beckman ultracentrifuge tubes, and samples were centrifuged at 30,000 rpm at 22°C for more than 48 h in a SW55 rotor. After centrifugation, the tube was inserted into a gradient collector, a hole was punctured at the bottom of the tube, and fractions of 5 drops each were collected in Eppendorf tubes (up to 50 fractions). The DNA concentration of each fraction was measured using a spectrophotometer, and the refractive index measured using a refractometer, after which the fractions were slot blotted onto a positively charged nylon membrane. The wells of the slot blotter were washed with denaturation buffer (0.5 M NaOH, 0.5 M NaCl). The membrane was then air dried and UV cross-linked. The HPV genomes were detected using DIG-labelled probes (see Southern blot analyses above).

## Results

### HPV16, but not HPV11 genomes, are maintained in keratinocytes during passage in tissue culture

**Fig 1:**
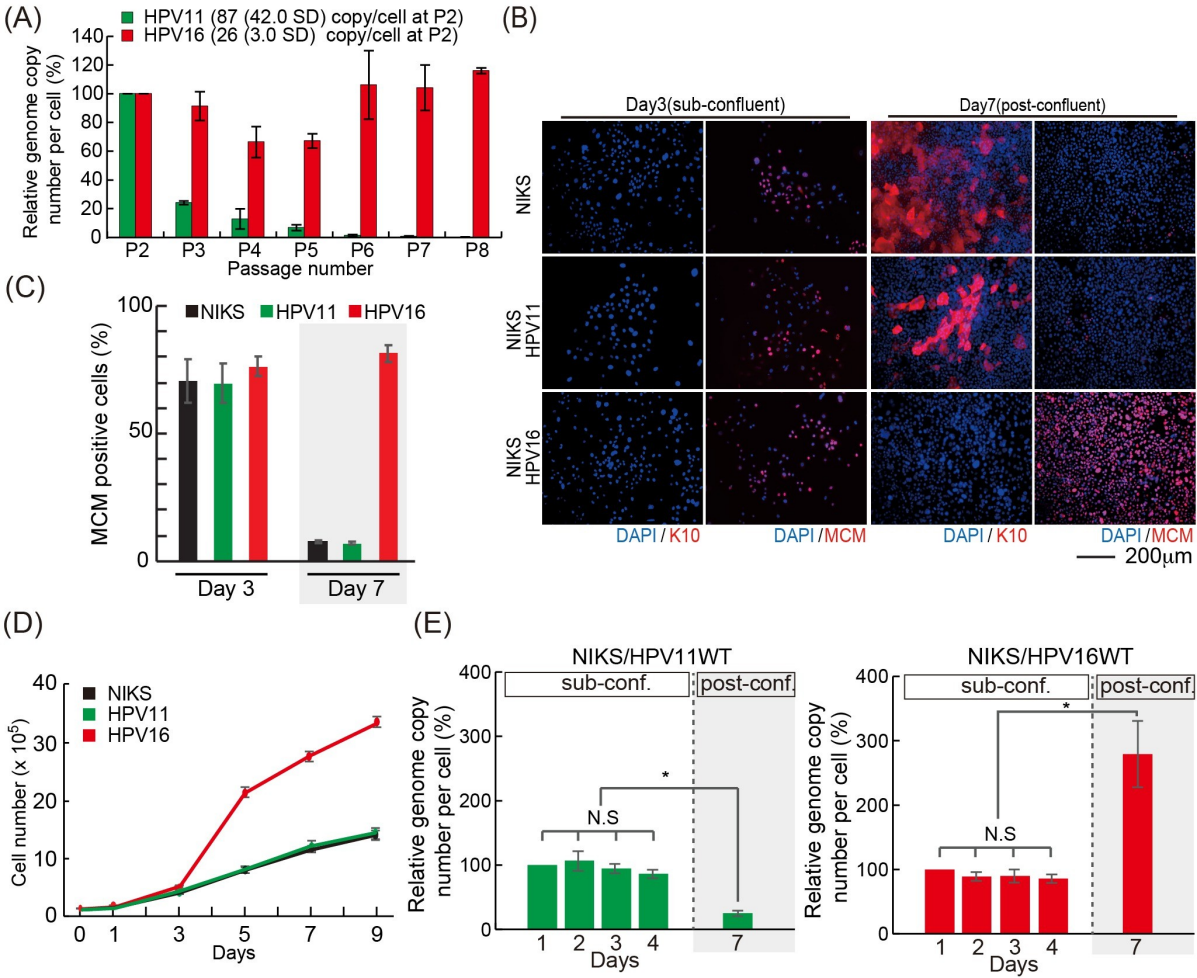
Difference between low and high-risk HPV types. (A) The viral genome copy number per cell was measured by qPCR, using the cellular GAPDH gene to estimate cell number. All DNA samples were digested with *Dpn*I to eliminate residual input DNA. The viral genome copy number per cell at passage 2 was set at 100%, and subsequent change in copy number normalized to this. HPV16 genome, but not HPV11, was well maintained until passage 8. The average viral genome copy number per cell at passage 2 is shown with standard deviation (SD). (B) NIKS or NIKS harboring HPV11 or HPV16 at days 3 and 7 were stained with K10 (red), to identify the early stage of differentiation; MCM (red), to identify the G1 and S phase compartments, and DAPI (blue) as a nuclear counterstain. Bar = 200μm. (C) Proportion of MCM positive cells at each time point is counted and shown. (D) NIKS, or NIKS harbouring HPV11 or HPV16 were counted in triplicate at days 1, 3, 5, 7 and 9 post-seeding. The cells were pre-confluent at day 3, confluent at day5 and post confluent at days7 and 9. (E) NIKS transfected with HPV11 (left) or HPV16 (right) were seeded at day 0, and collected at days 1, 2, 3 (pre-confluence), day 4 (confluence) and day 7 (post-confluence). The viral genome copy number per cell was measured as outlined in Fig 1A above. All DNA samples were digested with *Dpn*I to remove residual input DNA. The viral genome copy number per cell at each day 1 timepoint, was set at 100%. N.S. = no significant difference compared with each other. The single asterisk indicates P values of <0.01.

In order to compare the specific requirements for HPV16 and HPV11 genome replication in ‘infected’ basal-like keratinocytes we used NIKS cells, which are an isogenic immortal keratinocyte cell line previously shown to recapitulate the full epidermal differentiation program *in vitro* and to support the full HPV life cycle [22–24]. In order to establish NIKS cells harboring low- and high-risk HPV genomes, HPV11 and HPV16 genomes were transfected into NIKS cells along with a plasmid encoding a blasticidin resistance gene. Following drug-selection with blasticidin S, cells containing the HPV genomes were expanded and maintained at sub-confluent conditions for 2 to 8 passages. Total DNA was extracted at each passage and treated with *Dpn*I to remove the input genomes, and the levels of replicated viral genome were monitored by qPCR using GAPDH as a reference gene to calculate the HPV genome copy number per cell. As previously indicated [25–27], the HPV11 genome copy number rapidly declined upon passage to typically reach less than 0.2 copies/cell by passage 8 (Fig 1A). In contrast, HPV16 genomes, and indeed the genomes of other high-risk HPV types [23], persisted at a uniform copy number throughout the experiment. Although the actual HPV genome number varied between experiments, and was different for each HPV type, the trend was similar between passages. In order to overcome these tissue culture issues, and to carry out our experiments in a more controlled manner, we decided to monitor the viral genome copy number variations within the same passage (passage 2), growing cells from sub-confluent to post-confluent conditions in a 7 day time-frame. Previous studies have indicated that epidermal keratinocytes cultured *in vitro* switch between two interconvertible growth modes depending on the local cell density: an expanding growth mode, characterized by and excess of proliferating cells, and a balanced growth mode in which proliferation is counterbalanced by differentiation [28]. Similarly, as can be seen in Fig 1B (upper panels), NIKS cells grown in monolayer switch from a proliferative and undifferentiated (expanding) growth mode at sub-confluence (Day 3), in which they express the cell cycle progression marker MCM7 in the absence of the early keratinocyte differentiation marker keratin 10 (K10), to a differentiated and post-mitotic (balanced) growth mode at post-confluence (Day 7). Interestingly, when low passage NIKS harboring HPV11 and HPV 16 (passage 2 post-transfection/selection) were analyzed in a similar way, NIKS/HPV11 showed a very similar growth pattern to that of parental NIKS Fig 1B middle panels, Fig 1C and 1D), indicating that low-risk HPV fails to modulate keratinocyte homeostasis. In contrast however, while HPV16 did not significantly affect the growth mode of NIKS at sub-confluence, it conferred a potent proliferative capacity to post-confluent cells, which was accompanied by a loss of keratinocyte differentiation marker (Fig 1B lower panels, Fig 1C and 1D). This indicates that HPV16 is able to overcome the normal contact inhibition signals promoting the maintenance of an expanding growth mode at high cell densities. We then went back to analysing the HPV genome copy number in low passage (passage 2) HPV11/16 transfected NIKS, and found that both HPV11 and HPV16 have the same ability to maintain their genomes in sub-confluent keratinocytes (Fig 1D; days 1–4). At post-confluence (Day 7), however, HPV16 genome copy number undergoes an approximately 3-fold increase relative to day 4, while HPV11 genome copy number declined significantly (Fig 1E). When taken together, these data indicate that maintenance-replication of both low- and high-risk HPV types can be similarly efficient, but that HPV11 is unable to overcome keratinocyte differentiation at higher cell density, which compromises its replication ability. In all cases, viral genomes were episomal, as experiments were carried out shortly after transfecting circularized virus genome (confirmed by Southern blotting at passage 2 ([18], S1 Fig)). As predicted from this, transcript mapping studies revealed a characteristic ‘episomal’ pattern of viral gene expression in both HPV16 and HPV11-NIKS, with the majority of transcripts spanning E6 and E7, and terminating downstream of the E5 ORF[18].

### HPV 11 and HPV16 are replication competent in keratinocytes, with similar partitioning characteristics

**Fig 2:**
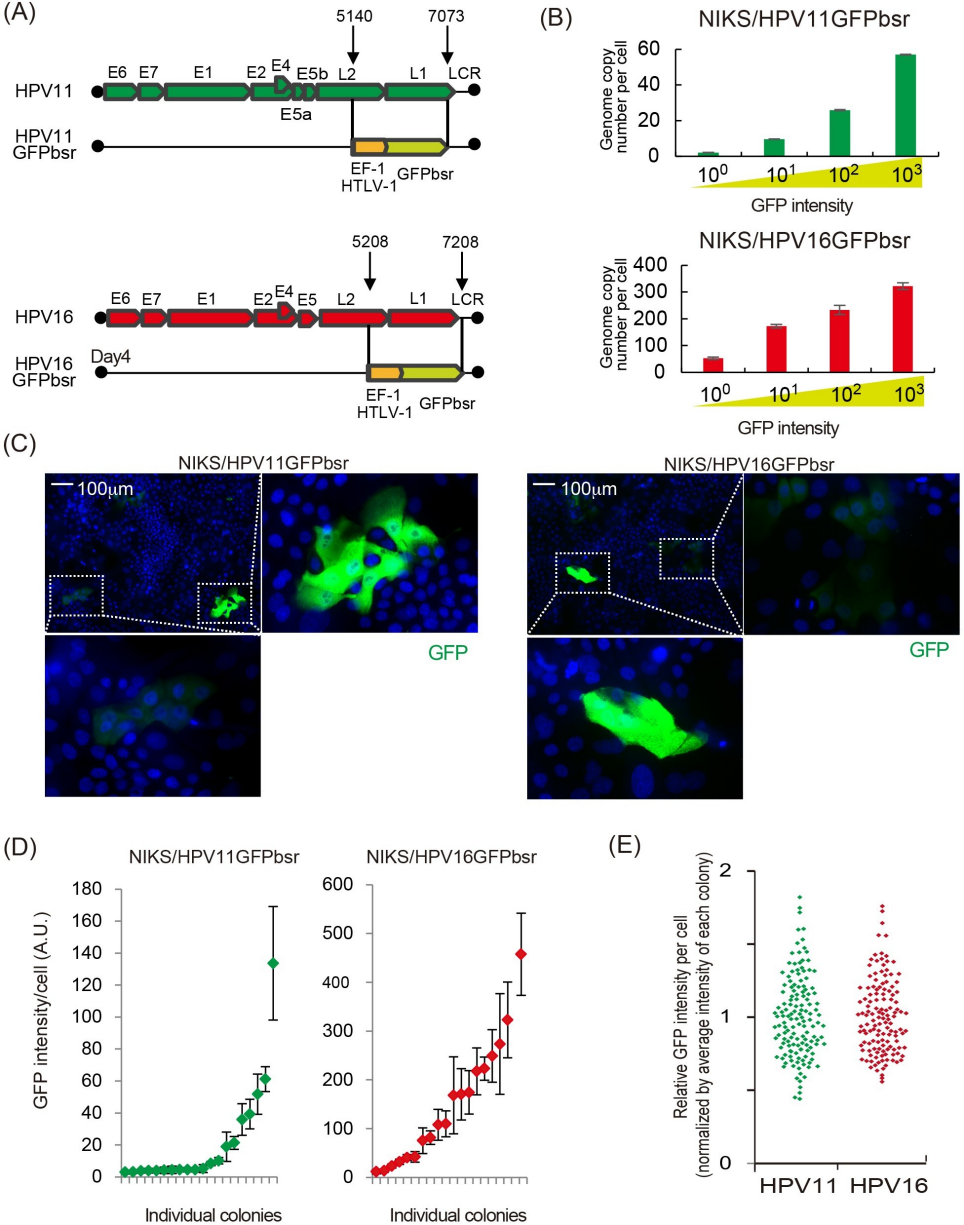
HPV11 and HPV16 are partitioned similarly in dividing NIKS cells. (A) The position of the hEF1-HTLV promoter-driven GFP-Blastocidin gene is shown in the context of the HPV11 and HPV16 genomes. The region between nt 5140 and 7073 is substituted by the GFPbsr cassette in HPV11GFPbsr, with a similar substitution between 5208 and 7208 in HPV16GFPbsr. (B) NIKS/HPV11GFPbsr or NIKS/HPV16GFPbsr cells were cultured to pre-confluence before FACS sorting into 4 groups on the basis of GFP intensity. Average HPV genome copy number in each group was determined by qPCR and is shown on the y-axis. (C) A uniform GFP intensity is seen in cells within NIKS/HPV11GFPbsr and NIKS/HPV16GFPbsr colonies following expansion in tissue culture. Enlarged images of the boxed colony are also shown. Colonies shown are representative of those analysed in (D). (D) NIKS/HPV11GFPbsr and NIKS/HPV16GFPbsr cells were plated along with parental NIKS, and colony size allowed to increase. 4 days later, the GFP intensity of cells within 20 HPV-positive colonies was investigated. For each individual colony, the green/red diamonds indicate mean GFP-intensity. Error bars show the extent to which GFP intensity varied in individual cells within each colony. (E) The individual GFP intensity of each cell was normalized to the mean GFP intensity of cells in its colony. Although individual colonies differed in their mean GFP intensity, the relative variation in GFP signal in individual cells within each colony was similar. Not obvious differences were apparent between HPV16 and 11.

As NIKS cells underwent 2 or 3 doublings during the first 4 days of our experiments, our data suggest that HPV11 does not differ from HPV16 in its overall replication competence, and can replicate along with cellular DNA in proliferating keratinocytes. Importantly, our results also suggest a similar ability of both genomes to partition accurately during cell division. To investigate this further, a hEF1-HTLV promoter/GFP-Blastocidin reporter cassette was inserted into the late gene region of HPV11 and HPV16 (Fig 2A) in order to directly visualize cells containing HPV genomes following transfection, an approach similar to the that used previously [29, 30]. The GFP-Blastocidin fusion gene also allowed the use of drug-selection to enrich for cells harbouring recombinant viral episomes. This is particularly important for low-risk HPV types such as HPV11, which does not obviously modify the cell phenotype, and has a tendency to decline in copy number over passage (Fig 1A and 1B). To examine whether the GFP intensity correlated with HPV copy number, NIKS cell populations harboring either the HPV11 or HPV16 reporter genomes (i.e. NIKS/HPV11GFPbsr or NIKS/HPV16GFPbsr, respectively), were separated by fluorescent activated cell sorting (FACS), into 4 groups according to the intensity of the GFP signal. Total DNA was subsequently extracted from each group, and HPV genome copy number was quantified by qPCR. In both the NIKS/HPV11GFPbsr and NIKS/HPV16GFPbsr populations, the intensity of the GFP signal correlated well with HPV genome copy number (Fig 2B). The NIKS/HPV11GFPbsr and NIKS/HPV16GFPbsr populations were then seeded along with parental NIKS at a ratio of 1:50. Colonies were allowed to expand in the dish for 4 days without drug selection, before being fixed and examined by fluorescent microscopy. Single GFP-positive cells were readily apparent immediately after plating, with larger GFP-positive colonies developing by day 4. Although there was great diversity in the GFP signal, suggesting great diversity in viral copy number, in individual colonies, individual cells within the same colony always showed uniform levels of fluorescence, irrespective of whether they contained HPV16 or HPV11 genomes (Fig 2C and 2D). In fact, when normalized to the mean GFP intensity of each colony, the variation in GFP intensity amongst individual cells in both NIKS/HPV11GFPbsr and NIKS/HPV16GFPbsr colonies was broadly similar (Fig 2E). When taken together, these results suggest that the HPV11 genome is partitioned with similar dynamics to that of HPV16 in proliferating keratinocytes.

### Increased cell density mediates a switch from E1-independent to E1-dependent replication

**Fig 3:**
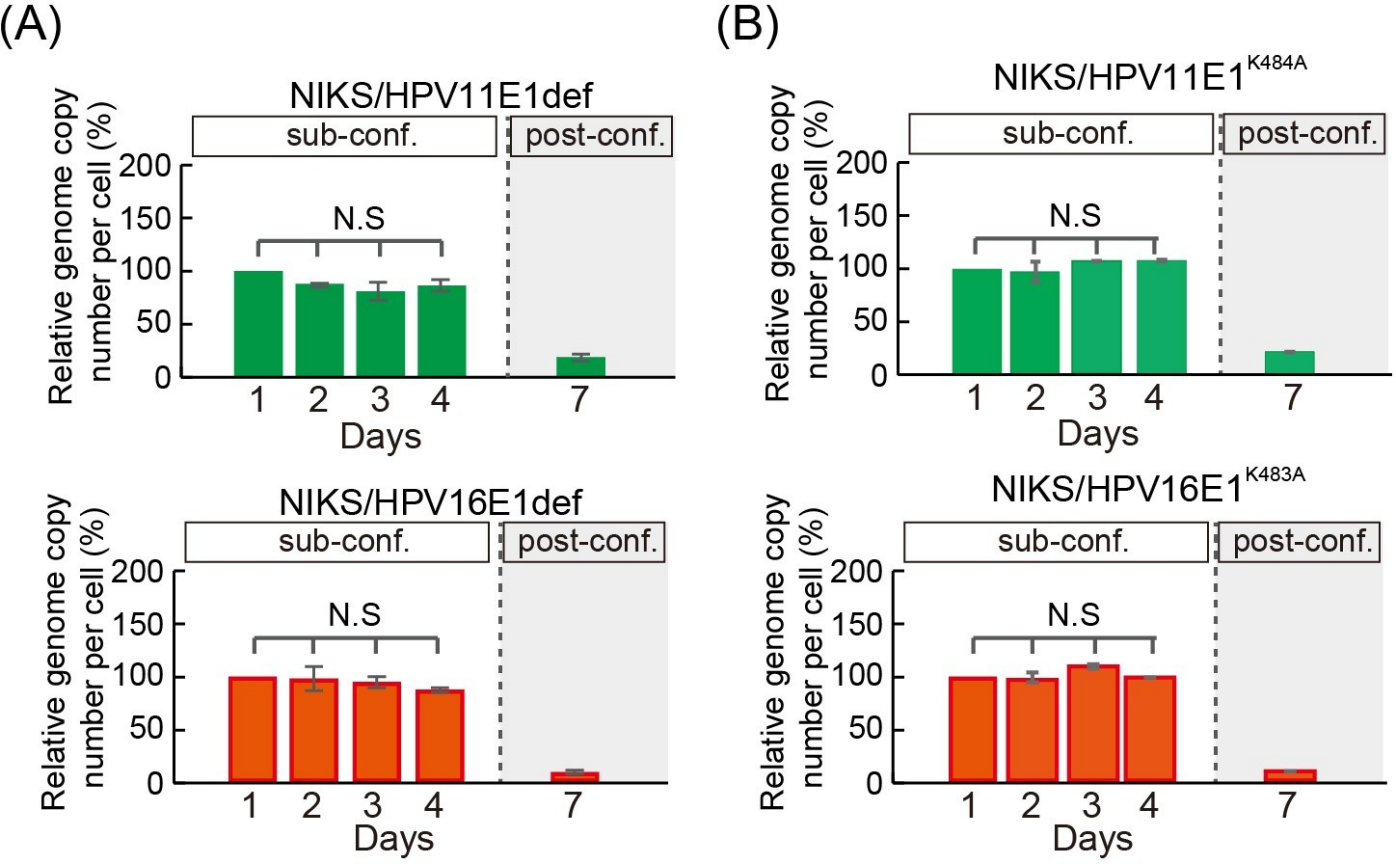
Requirement of a helicase-active E1 for genome amplification but not maintenance. (A) The virus genome copy number per cell of E1-defective HPV11 (HPV11E1def, upper) and HPV16 (HPV16E1def, lower) transfected NIKS was measured, and prepared for presentation as outlined in Fig 1E. (B) The virus genome copy number per cell of E1 helicase-defective HPV11 (HPV11E1^K484A^ left) or HPV16 (HPV16E1^K483A^, right) transfected into NIKS was measured and prepared for presentation as outlined in Fig 1E. Both the HPV11 E1^K484A^ and HPV16 E1^K483A^ genome declined post-confluence in the absence of a helicase-active E1 gene.

Following papillomavirus infection of basal keratinocytes, the productive phase of the virus life cycle is triggered by host keratinocyte differentiation, with viral genome replication and amplification rising gradually as cells enter the suprabasal layers, followed eventually by the expression of viral capsid proteins and virion assembly in the uppermost layers of the epithelium [31, 32]. The fact that HPV11 is unable to maintain or amplify its genome during the commitment to differentiation in our experimental setting (Fig 1B and 1E), indicates that the modulation of keratinocyte differentiation is crucial for genome maintenance, and that differentiation-commitment can negatively influence the HPV replication machinery. During productive infection, the viral DNA helicase E1 is essential for genome amplification in the mid epithelial layers [7], whereas the importance of E1 for maintenance-replication in basal keratinocytes is still controversial [17, 33]. This data suggests that HPV might follow a ‘two-phase’ replication mode with respect to E1-dependency [34]. To establish the contribution of E1 to HPV16/11 maintenance-replication, as well as for HPV16 genome amplification in differentiating keratinocytes, E1 deficient mutants were generated in the context of the full length HPV11 and HPV16 genomes (HPV16E1def & HPV11E1def), and stable NIKS populations were established as described above. At the time of seeding for the experiment, (passage 2 post-transfection/selection) viral genomes were predominantly episomal as determined by Southern blotting (S1 Fig). The viral genome copy number was monitored to see its variations within the same passage in the same way as shown in Fig 1E). As can be seen in Fig 3A, both HPV11E1def and HPV16E1def genomes were maintained at similar levels as the WT genomes until day 4 during the ‘maintenance-replication’ phase, but declined dramatically post-confluence (Day 7). These data support the hypothesis that HPV16 at least, can switch its replication mode from E1-independent to E1-dependent replication as cell density increases, and that HPV16 does not simply hamper transit to a balanced growth mode at high cell density and prolong the expanding growth mode as well as the maintenance replication of virus genome. In addition, these results show that both HPV11 and HPV16 can replicate without the E1 viral helicase in expanding NIKS cells, and that the inability of low-risk HPV to overcome differentiation signals triggered by high cell densities has dramatic consequences on their replication capacities. To support these conclusions, a second set of E1 defective mutants were made in the context of the HPV16 and 11 genomes, this time to disrupt the integrity of the conserved E1 helicase domain that is essential for E1’s replicative function (HPV11 E1^K484A^ and HPV16 E1^K483A^)[35]. The results obtained with these mutants were indistinguishable from those seen with HPV11E1def and HPV16E1def mutants (Fig 3B).

**Fig 4:**
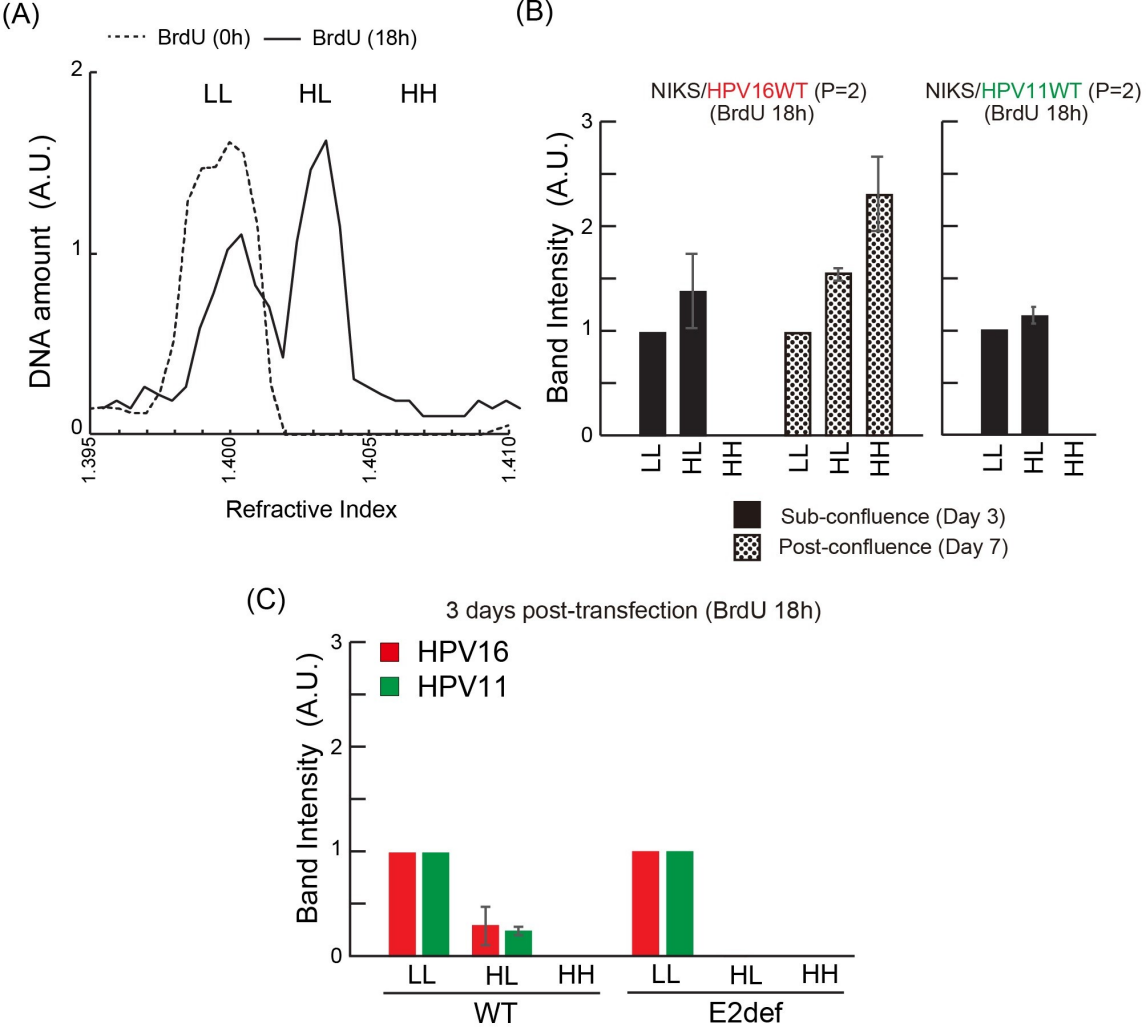
Synchronous HPV DNA replication durind sub-confluent growth. (A) Profile of NIKS cellular DNA 18 h after BrdU labelling. The DNAs were separated on a CsCl gradient, and fractions were collected. In each fraction, refractive index (RI) and DNA concentration were measured by a spectrophotometer and a refractometer respectively. The positions of heavy-heavy DNA (HH), heavy-light DNA (HL), and light-light DNA (LL) are marked. (B) DNA of NIKS containing HPV16 or 11 was collected at day 3 and 7 of passage 2 following 18 h of BrdU labelling. The DNAs were separated on a CsCl gradient, and fractions were collected as before. Fractions where the RI indicated the possible presence of DNA (HH, HL or LL) were blotted, before the detection and quantitation of HPV DNA using DIG-labelled probes. (C) DNA from NIKS containing HPV16 or 11 genomes was collected at 3 days post-transfection following 18 h of BrdU labelling. HH, HL or LL HPV DNA was separated and detected as described in (B).

To determine the two modes of HPV DNA replication in different cell density, we investigated how many times HPV DNAs replicate in a single S phase by labelling cells with BrdU [26, 27, 34]. As Hoffman et al. reported, DNAs that have replicated once will incorporate BrdU on a single DNA strand (heavy-light, HL). Those that have replicated more than once will incorporate BrdU on both strands (heavy-heavy, HH), and those that didn’t replicate will have no BrdU in their DNA (light-ligh, LL). These three DNA species were separated on a cesium chloride gradient according to their different densities, with LL DNA at 1.709 g/ml (Refractive Index (RI) = 1.400), and HL DNA at 1.753 g/ml (RI = 1.404) and HH DNA at 1.795 g/ml (RI = 1.708) (Fig 4A and 4B). Both HPV16WT and HPV11WT genomes were replicated once in a single S-phase, with the emergence of HL viral DNA, but not HH viral DNA at day 3 during the E1-independent maintenance replication phase (Fig 4B). In contrast, the HPV16 WT genome was replicated more than once at day 7, during the E1-dependent replication phase, with the emergence of HH viral DNA at day 7 (Fig 4B). These results suggest that the mode of HPV genome replication switches from a synchronous mode (i.e. once in a single S-phase), to amplification mode (i.e. multiple times in a single S-phase) in a cell-density related manner. During the establishment of infection, it is thought that one single viral particle may efficiently establish the full viral intracellular life cycle [36], suggesting that the papillomavirus DNA copy number is amplified to a certain level (50 to 400) per cell immediately after infection. Prior to P1, at 3 days post transfection, both HPV16WT and HPV11WT genomes were replicated once in a single S-phase, with the emergence of HL viral DNA but not HH, with only a small proportion of total HPV DNA contributing to replication (Fig 4C). At this point the majority of the viral DNA extracted from the NIKS cells is *DpnI* sensitive (91.2% (1.6 standard deviation)). Interestingly, both the HPV11 and 16 E2 defective genomes were not successfully replicated, with no HL and HH viral DNA produced (Fig 4C). These results suggest that in our experiments during the establishment phase, HPV genomes are replicated once per S-phase in an E2-dependent manner, after the introduction of multiple copies of viral DNA by transfection. We suspect that the initial amplification phase that occurs after infection by a single virus, and which is thought to be E1-depenent, is bypassed as a result of the introduction of a high copy number of episomal viral DNA by transfection.

### HPV11 genome is replicated post-confluence in the presence of HPV16 E6 and E7

**Fig 5:**
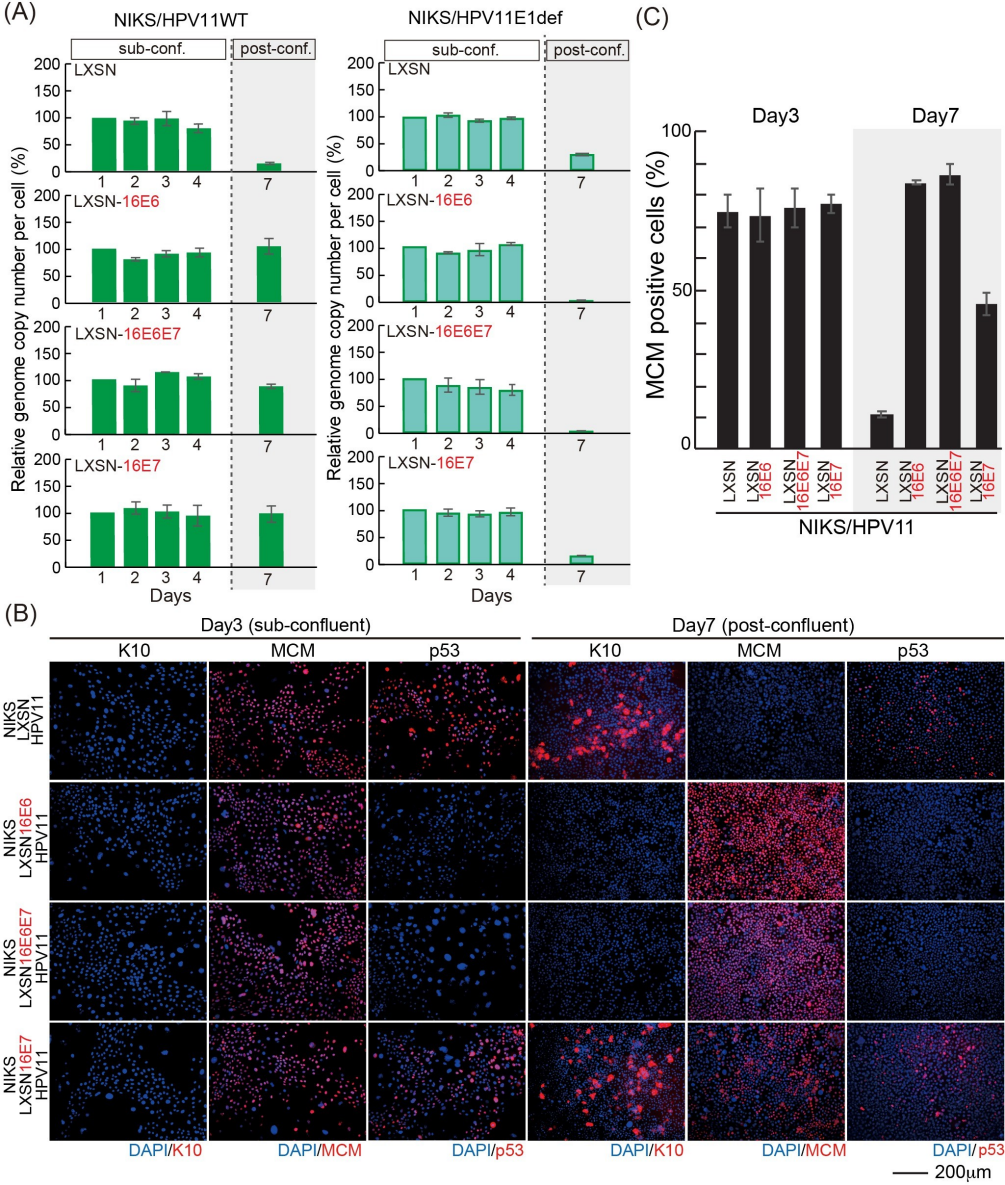
Low-risk HPV is maintained in NIKS expressing high-risk HPV E6 and E7 post-confluence. (A) The virus genome copy number per cell of HPV11 (left) and HPV11 E1def (right) transfected into NIKS or NIKS expressing HPV16 E6 and/or E7, was measured and prepared for presentation as outlined in Fig 1E. (B) NIKS or NIKS expressing HPV16 E6 and/or E7 at days 3 and 7 were stained with K10 (red); MCM (red), p53 (red), and DAPI (blue). Bar = 200μm. (C) The proportion of MCM positive cells shown in (B) is quantified and shown.

Although E1 is dispensable for the maintenance of either HPV11 or 16 genomes in dividing NIKS cells, it is clearly important for the HPV16 genome copy number elevation in post-confluent keratinocytes, with E1 disruption leading to HPV16 genome loss as cells transition to post-confluence. The different abilities of HPV11 and HPV16 to replicate their genomes in keratinocytes at high cell density prompted us to investigate the interplay between the viral replicative machinery and the HPV accessory proteins E6 and E7. Recent studies indicate that HPV16 E6 has a predominant role, over E7, in skewing basal keratinocyte cell fate towards proliferation by inhibiting differentiation in a p53- and Notch-dependent manner [20, 37]. In order to assess the potential roles of E6 and E7 functions in creating a replication-competent environment for the virus, we tested whether high-risk HPV E6/E7 proteins might complement the replicative deficiency of HPV11 at high cell densities. To do this, we established NIKS cells stably expressing HPV16 E6 and/or E7 by retroviral transduction, and the expression of E6 and E7 were confirmed by western blot analysis (S2A Fig). The HPV11 genome was transfected into these cell lines and NIKS transduced with empty vector (LXSN) as a control, and, following drug selection, cells were seeded at passage 2 or 3 post-transfection for the evaluation of the viral genome copy number. Strikingly, the expression of HPV16 E6 and/or E7 allowed replication of the wild-type HPV11 genome post-confluence (Fig 5A left panel), which was associated with an inhibition of differentiation and p53 expression when HPV16 E6 was expressed either alone or in combination with E7, as well as a potent increase in the cell-cycle activity post-confluence (Fig 5B and 5C). It is interesting to note however, that unlike E6, HPV16 E7 exerts its function by uncoupling cell-cycle entry from differentiation as previously reported [38]. In order to confirm that the expression of the viral helicase E1 was indeed necessary to mediate post-confluence HPV11 genome maintenance in the presence of HPV16 E6 and E7, we transfected the E1-defective HPV11 genome in NIKS expressing HPV16 E6 and/or E7 and the viral genome copy number was monitored across 7 days. The results of this assay are shown in Fig 5A (right panel), and as can be seen, the lack of a functional E1 gene product impaired the ability of HPV16 accessory proteins to rescue HPV11 genome replication post-confluence. Taken together, these data indicate that there is an interplay between the replication and accessory proteins of HPV, and that disruption of this interplay affects genome maintenance as cells transit towards a balanced growth mode and E1-dependent replication is triggered at high cell density. Our data suggest that maintenance replication in dividing basal cells prior to this may be E1-independent.

### The inactivation of p53 is required for viral genome replication post-confluence in both HPV11 and HPV16, but not for maintenance replication in sub-confluent cells

**Fig 6:**
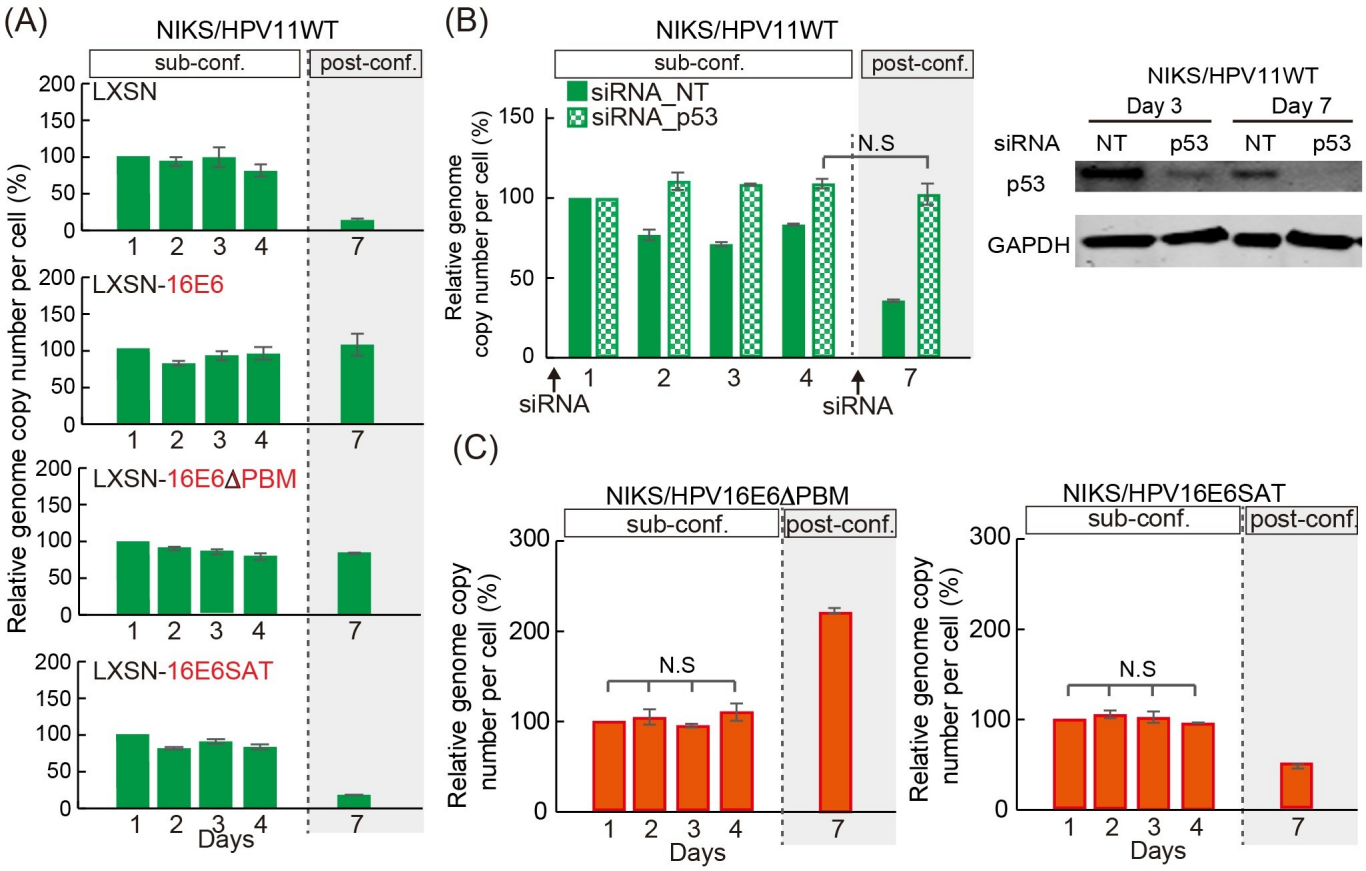
Inactivation of p53 rescues genome maintenance of low-risk HPV post-confluence. (A) The virus genome copy number per cell of HPV11 transfected NIKS or NIKS expressing 16E6, HPV16 E6ΔPBM (16E6ΔPBM) or HPV16 E6SAT (16E6SAT) was measured and prepared for presentation as outlined in Fig 1E. (B) The viral genome copy number per cell of HPV11 in NIKS transfected with siRNA (control (NT) or to p53) was carried out as in Fig 1E. Cells were collected at day 3 and 7 to establish p53 levels in the different cell populations (right). (C) The full genome of HPV16E6ΔPBM (left) or HPV16E6SAT (right) was transfected into NIKS. The viral genome copy number per cell was measured and prepared for presentation as outlined in Fig 1E.

Previous studies indicate that the ability of HPV16 E6 to target p53 and PDZ domain-containing proteins for degradation is required for the successful completion of the viral life cycle [16, 39–41]. Our result showing that 16E6 is able to facilitate HPV11 replication post-confluence, prompted us to investigate what functions of the 16E6 protein are essential to mediate this effect. To do this, we established two NIKS cell lines by retroviral transduction, to express either the HPV16 E6 SAT mutant (NIKS/LXSN-16E6SAT), which is unable to bind and target p53 for degradation, or the PDZ-binding defective mutant (NIKS/LXSN-16E6ΔPBM), which lacks the C-terminal PDZ-binding motif (PMB) responsible for the association with PDZ proteins. The wild-type HPV11 genome was transfected into these cell lines, and genome replication was assessed in sub- and post-confluent cells. As expected, neither of the 16E6 mutants affected HPV11 genome maintenance sub-confluence (Fig 6A), with the loss of p53-degredation capability alone, affecting E6’s ability to support viral genome replication post-confluence (Fig 6A). In order to further examine the importance of p53-loss on genome maintenance as cells become confluent, the expression of p53 was transiently ablated by RNA interference (siRNA) in NIKS cells harboring wild-type HPV11 genomes, and the effects on genome maintenance and replication are shown in Fig 6B. As can be seen, the levels of p53 were efficiently reduced both in sub- and post-confluent cells (Fig 6B lower panel), and consistent with this, a higher level of maintenance replication was seen in sub-confluent cells from days 2 to 4 (Fig 6B upper panel). Intriguingly, the ablation of p53 had its most dramatic effects post-confluence, where cells transfected with the control non-targeting (NT) siRNA showed the most dramatic decline in HPV11 copy number (Fig 6B upper panel). These results suggest that p53 binding, but not the association with PDZ domain-containing proteins, is necessary and sufficient to allow HPV genome maintenance in confluent keratinocytes. In order to confirm that a similar role for E6 during HPV16 replication, we engineered the wild-type HPV16 genome to express either an E6 protein defective in its PDZ-binding activity (E6ΔPBM), or the E6SAT mutant that is unable to interact and degrade p53. Mutant genomes were transfected into NIKS, and after selection, low passage NIKS transfectants were seeded and viral genome replication assessed. As can be seen in Fig 4C, both HPV16E6ΔPBM and HPV16E6SAT mutant genomes were replicated until day 4, but the copy number of HPV16E6SAT mutant genome dramatically declined post confluence at day 7 (Fig 6C, right). In contrast, the HPV16E6ΔPDZ mutant genome was amplified as efficiently as the wild-type genome (Fig 6C, left). These results suggest that E6-mediated p53 inactivation contributes to genome maintenance as cells reach confluence, and point perhaps to a more important role in this than for E7.

Previous studies have shown E6 and E7, but not E4 and E5, to be required for the establishment and maintenance of HPV genomes in keratinocytes in a tissue culture model [18, 25, 42–44]. Our results suggest that E1, and some of 16E6 functions (degradation of p53 and PDZ-proteins) are not required for replication in growing keratinocytes. To address this further, E6 and E7 deficient mutants in the context of HPV11/16 genomes, were transfected into these cell lines, and the viral genome copy number monitored over 7 days. As can be seen in S3A and S3B Fig, both E6 and E7 defective genomes of HPV11 and 16 were established and maintained at levels similar to those of the WT genomes until day 4 during the ‘maintenance-replication’ phase, but declined dramatically post-confluence (Day 7). This data supports the idea that E6 and E7, the viral accessory genes, are required only for E1-dependent replication but not for E1-independent replication.

Although most of the HPV11 and HPV16 mutant genomes replicated successfully in sub-confluent NIKS cells (with the exception of the E2 knock out), we have not been able to establish long term cultures of NIKS cell lines harboring any of these HPV genomes, which is consistent with our earlier studies on cell passage, as well as with previous reports [16, 40]. As HPV16SAT and HPV16E1def genome copy numbers also declined during passage, we conclude that E1 and the inhibition of p53 are required for long-term tissue culture maintenance of HPV16 genomes (S4A Fig). Interestingly, the ability of 16E6 to target PDZ proteins, as well as the presence of E7, was also required for the genome maintenance during cell passage in this system (S4A Fig) [16]. Previous reports suggest that the stress induced by cell passaging triggers keratinocyte differentiation via activation of the Notch pathway [45], indicating an overlapping mechanism of viral genome loss during passaging and at high cell densities, where E1-dependent replication could be triggered by the induction of keratinocyte differentiation. The fact that HPV11 WT genomes were not maintained over passage also in the presence of HPV16E6 and E7 (S4B Fig), suggests that the requirements for copy number maintenance during passage are more stringent than those that affect cells at and around the point of confluence. Our focus here is on the latter, given that this is the more physiologically relevant situation.

### HPV11 E6 is able to stimulate p53 degradation in a cell density-dependent manner and support the replication of HPV11 post-confluence

**Fig 7:**
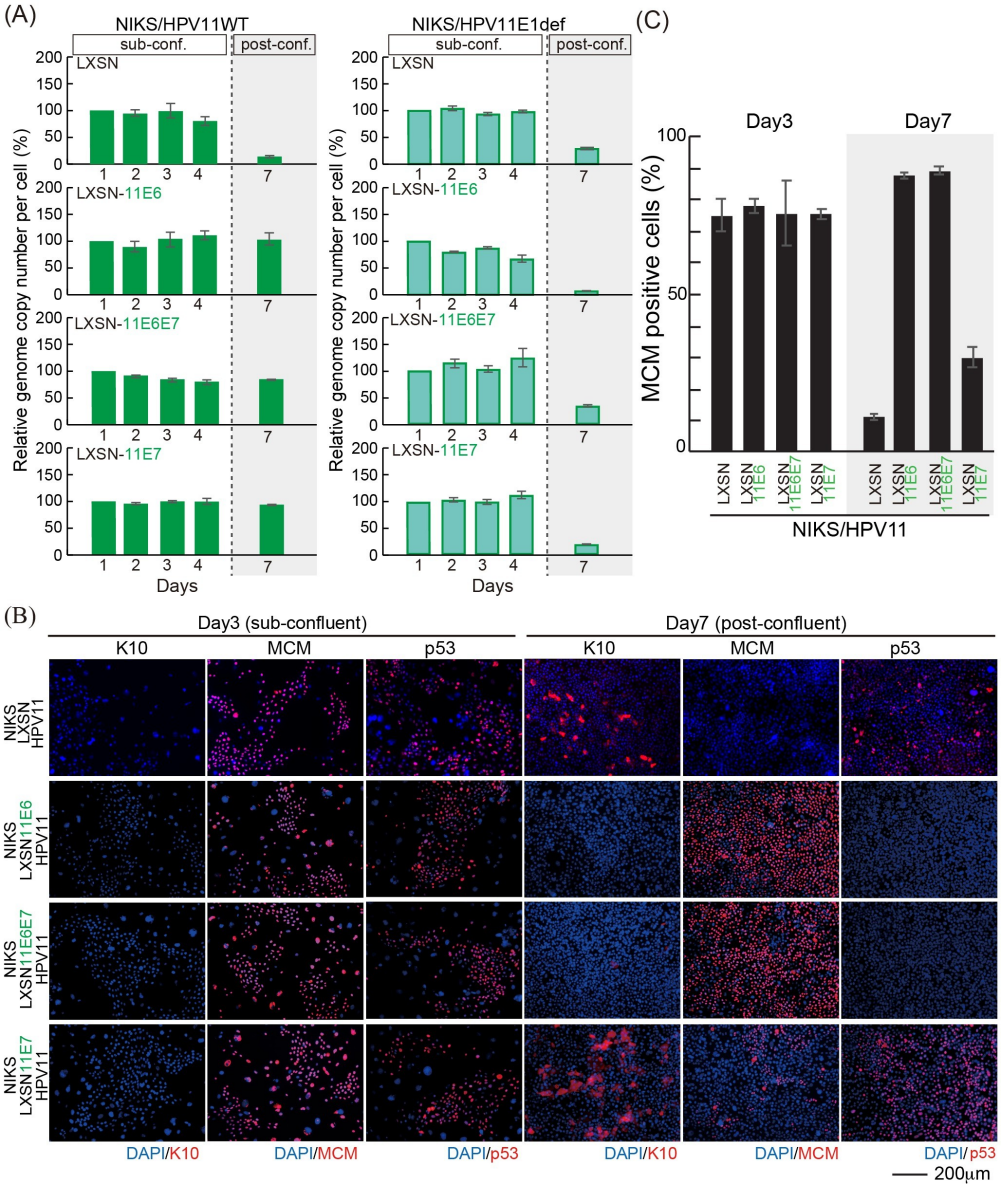
Low-risk HPV is maintained in NIKS expressing low-risk HPV E6 and E7 post-confluence. (A) The virus genome copy number per cell of HPV11 (left) and HPV11 E1def (right) transfected into NIKS or NIKS expressing HPV11 E6 and/or E7 was measured and prepared for presentation as outlined in Fig 1E. (B) The NIKS and NIKS expressing HPV11 E6 and/or E7 at day 3 and 7 were stained with K10 (red), MCM (red), p53 (red) and DAPI (blue). Bar = 200μm. The images of NIKS at day 3 and 7 were shown previously in Fig 3C. (C) The proportion of MCM positive cells shown in (B) is quantified and shown.

**Fig 8:**
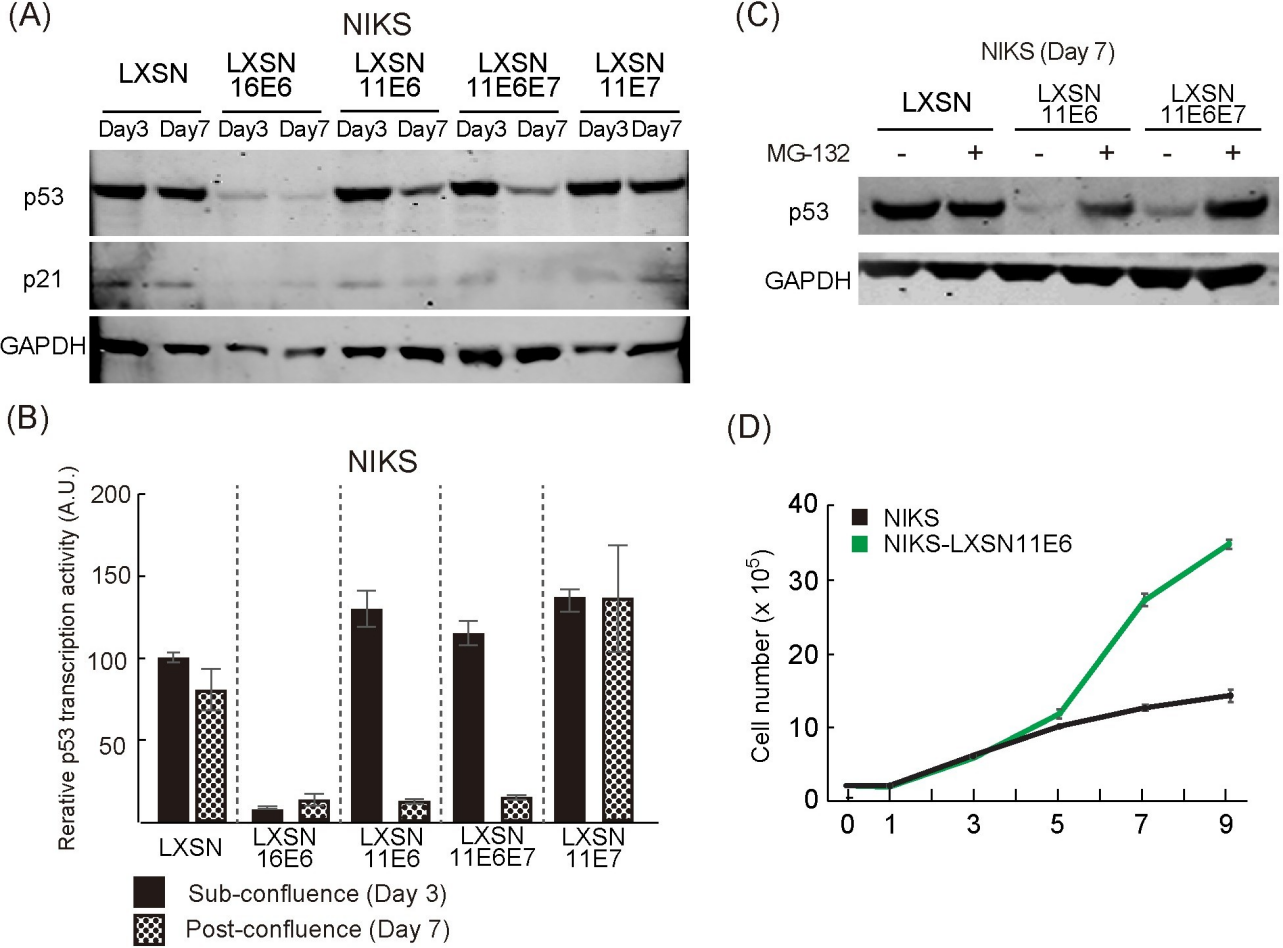
HPV11 E6 drives a reduction in p53 levels only at post-confluence. (A) Whole cell extracts of NIKS or NIKS expressing 16E6, 11E6, 11E6/E7 and 11E7 at day 3 and 7 were used to assess the levels of p53 and p21 by western blot. (B) A p53 reporter assay was performed to assess p53 transcriptional activity. Each assay was carried out either at day 3 and 7 using NIKS or NIKS expressing 16E6, 11E6, 11E6E7 and 11E7. The activity of firefly luciferase normalized using the activity of Renilla luciferase. The p53 transcriptional activity of NIKS at day 3 was set at 100%, and other measurements normalised to this. (C) 1nM of the proteasome inhibitor, MG-132, or an equal volume of the carrier (dimethyl sulfoxide) was added to NIKS or NIKS expressing 11E6 or 11E6E7, and incubated for 3 hrs. Whole cell extracts were used to assess the level of p53 by western blotting. The loss of p53 protein was restored by proteasome inhibition. (D) Elevation in growth rate in NIKS expressing 11E6, in comparison with the parental NIKD-LXSN cells. 11E6 has similar effects on cell growth, particularly post-confluence.

Previous studies have shown that HPV11 can inhibit p53 transactivation activity in an E6AP-independent manner through association with the p300 acetyl-transferase/transcriptional co-activator [46, 47], but have not yet revealed a p53 degradation capability comparable to that of HPV16. To understand HPV maintenance regulation further, viral transcripts from NIKS/HPV11 and NIKS/HPV16 cells grown to sub-confluence (Day 3) or post-confluence (Day 7), were examined following cDNA synthesis and qPCR. Interestingly, the level of the early promoter activity of HPV11 (p90) was very low when compared with HPV16 (p97) especially at day 7 (typically around 200 and 10500 copies per 1000 GAPDH mRNA copies respectively across repeat experiments). The expression level of transgenes from LXSN retrovirus vector is between 6000 and 12000 copies per 1000 GAPDH, which is broadly equivalent to the transcriptional activity of HPV16 early promoter. This result suggests that NIKS cells grown in monolayer may not support the expression of HPV11 E6 and E7 at levels high enough to allow viral genome replication post-confluence. To evaluate whether HPV11 E6 and E7 could have a similar effect to their HPV16 counterparts in mediating the replication of HPV11 genome post-confluence, we established NIKS cells stably expressing HPV11 E6 (NIKS/LXSN-11E6) and/or HPV11 E7 (NIKS/LXSN-11E6E7 and NIKS/LXSN-11E7) by retroviral transduction. After confirming the expression of HPV11 E6 and E7 in these cell lines by RT-PCR (S2B Fig), control (LXSN) and HPV11 E6 and/or E7-expressing NIKS were further transfected with the HPV11 genome, and the levels of viral replication were assessed by qPCR as described above. The HPV11 genome was again well maintained until day 4 in control NIKS, as well as in the presence of 11E6 and/or 11E7 during this ‘maintenance replication’ phase (Fig 7A left panel). As seen with HPV16 E6 and E7 however, the HPV 11 E6 and E7 proteins could also facilitate the replication, albeit not the amplification, of the HPV11 genome post-confluence. Furthermore, the lack of a functional HPV11 E1 gene impaired this post confluence maintenance ability, which appears to be mediated by a switch to E1-dependent replication (Fig 7A left panel). These results suggest that HPV11 E6 and E7 are similar to the HPV16 proteins in supporting HPV11 genome replication in post-confluence cells providing that they are present at sufficiently high levels. Having shown that HPV11 E6 and E7 are functionally equivalent to the high-risk HPV accessory proteins in supporting viral genome maintenance, we were interested in understanding how HPV11 E6 and E7 may achieve this. To do this, freshly transfected low passage control NIKS/LXSN harboring HPV11 genomes, and NIKS/HPV11 expressing HPV11 E6 and/or E7 were seeded, and the levels of MCM7, p53 and keratin 10 were assessed by immunofluorescence at sub- and post-confluence, similar to the experiments carried out using the HPV16 E6 and E7 proteins earlier (Fig 5). As shown in Fig 7B and 7C, and consistent with previous reports [48], at sub-confluence HPV11 E6 and E7 failed to significantly increase the levels of cell-cycle entry of NIKS or to promote the degradation p53. Surprisingly however, in post-confluent cells, HPV11 E6 and E7 showed very similar effects to those seen earlier with HPV16 E6 and E7 (Fig 7B and Fig 5); with HPV11 E6 leading to a loss of p53, an increase in the number of cycling cells, and an inhibition of commitment to differentiation at high cell densities. As mentioned previously, the level of the early promoter activity of HPV11 (p90) was not sufficient to show these phenotypes in this experimental model (Figs 5 and 7B and S5 Fig). Like HPV16 E7, HPV11 E7 was found to uncouple differentiation from proliferation (Fig 7B and 7C). To our knowledge, this is the first demonstration that both HPV11 and HPV16 E6 can similarly reduce p53 levels in the cell, albeit for 11E6, in a cell density-dependent manner. As a result of this, we were interested to establish whether the HPV11 E6-mediated loss of p53 in post-confluent cells occurs through a mechanism similar to that used by HPV16 E6. First we confirmed the immunofluorescence data by monitoring the levels of p53 in the presence of HPV11 E6 and/or E7 at sub- and post-confluence by Western blotting analysis, using HPV16 E6 as a positive control. As can be seen in Fig 8A, the expression of HPV16 E6 in NIKS cells led to a potent degradation of p53 at both time points, which also resulted in an inhibition of expression of the p53 target gene p21. Conversely, HPV11 E6, when expressed either alone or in combination with HPV11 E7, led to a reduction of p53 and p21 levels, but in this case, p53 loss was restricted to the post-confluent cell populations. As expected, HPV11 E7 had no influence on p53 levels in the rich culture medium conditions used at either day 3 or 7. The cell density-dependent inhibition of p53 transcriptional activity that is suggested from the p21 western blots described above, was further confirmed using a reporter gene assay, in which luciferase was expressed from a plasmid-containing tandem repeats of p53 responsive DNA elements (Fig 8B). In support of this, HPV11 E6 strongly affected p53 transcriptional activity only at high cell densities, whereas HPV16 E6 did so irrespective of the cell confluence status. Since HPV16 degrades p53 in an E6AP- and proteasome-dependent manner [49], we tested whether HPV11 E6 could also use this mechanism of p53 degradation in post-confluent cells. To do this, control (LXSN) NIKS cells or NIKS expressing HPV11 E6 alone or in combination with E7 were grown post-confluence and treated with the proteasome inhibitor MG-132 for an additional 3 hours prior cells were harvested and the levels of p53 monitored by Western blotting analysis. As can be seen in Fig 8C, our proteasome inhibition had no effect on p53 levels in control cells. In post-confluent NIKS expressing HPV11 E6 and E6/E7 the levels of p53 were strongly reduced however, with proteasomal inhibition leading to a potent up-regulation of the p53. Interestingly the 11E6 mutant (W133R), which cannot bind to E6AP [25, 50], still reduced p53 level at post-confluence (S6 Fig), suggesting that 11E6 mediates p53 degradation in an E6AP-independent manner. This conclusion is supported by a complimentary experiment showing that 11E6 is also able to reduce p53 abundance in the presence of shRNA against E6AP at post-confluence (S6 Fig). As expected from these results, HPV11 E6 conferred a potent proliferative capacity, especially on post-confluent cells, which was similar to what was seen with HPV16 E6 (Fig 8D). From this we conclude that HPV11 E6 is able to exploit the proteasomal pathway in order to target p53 for degradation, and suggest that this is regulated in the epithelial basal layer to support virus genome maintenance in response to increasing cell density and the eventual need to differentiate and enter the virus productive cycle.

## Discussion

The study of papillomaviruses has for the most part, focused on the association of high-risk Alpha papillomavirus types with anogenital and oropharyngeal cancers, and as a result of this, our knowledge of these types is more complete than our understanding of the low-risk Alpha papillomaviruses. The functional differences between low- and high-risk gene products, especially those encoded by the E6 and E7 accessory genes, provides a molecular explanation for the cancer-causing properties of the high-risk HPV types (see [6, 11, 39]). The low-risk types have however been as evolutionarily successful as the high-risk HPVs, and with respect to the molecular evolution of alpha HPVs and niche adaptation, their life cycle strategies centre around a basic set of molecular functions that are required by all alpha HPVs to complete their productive life cycle, with high-risk types having evolved additional functions which confer their oncogenic properties. The similarities, as well as differences between high- and low-risk papillomaviruses, particularly in the context of the virus life cycle, have not yet been elucidated in any detail. In this study, we have compared the replication capabilities of the two most representative members of the high-risk (HPV16) and low-risk (HPV11) alpha papillomavirus groups in a common keratinocyte background, and have analyzed the fluctuation in genome copy number over sequential passage, as well as day by day within a single passage. In contrast to HPV16, HPV11 was poorly maintained over passage, which is in agreement with previous reports [25, 51]. To our surprise however, both the HPV16 and HPV11 genomes replicated similarly in proliferating sub-confluent keratinocytes within the same passage, with the tendency towards keratinocyte differentiation as cell density increases, representing a crucial regulatory point that HPVs must control if their genomes are to be maintained long term. This modulation reflects a requirement for HPVs to subtly disturb normal epithelial homeostasis to favour retention of the infected cell in the basal layer [20]. At the point where keratinocytes transit to the balanced growth mode as cell density increases, the HPV replication mode appeared to switch from being an E1-independent/E2-dependent synchronised mode to an E1-dependent unsynchronised mode. At the same time, keratinocyte differentiation was suppressed by E6, with cell cycle re-entry (for genome amplification) being mediated by E6 and E7 (Figs 3C and 5B). In contrast to HPV16, where viral gene expression and the phenotypic consequences for the cell can be understood, for HPV11 only low levels of viral gene expression were apparent, which may cause low level modulation of p53 level (Fig 6B, right panel), but was not sufficient to show inhibition of differentiation and promotion of cell cycle at post-confluence (Figs 5B and 7B). When the HPV11 E6 and E7 proteins were expressed exogenously from a retroviral vector however, they had very similar effects on the cell as seen with 16 E6 and E7, with E6 promoting the proteasome-mediated degradation of p53 and inhibiting keratinocyte differentiation, which is thought to take place in the basal and/or parabasal layers, and E7 uncoupling cell-cycle entry from differentiation, which is thought to take place in the parabasal and upper layers. Of some importance however, was our observation that the HPV11 E6 protein was able to promote the degradation of p53 in a cell density-dependent manner via E6AP independent manner, which suggests that the mechanism of p53 regulation by low-risk HPV types is more subtle than that of the high-risk types. It is interesting to note however, that in both HPV11 and HPV16, E1 function as well as p53 inhibition were dispensable for viral genome replication up until the point that cells were triggered towards differentiation at confluence. These two phases in the virus replication cycle are intimately linked to the biology of the differentiating keratinocytes infected by the virus. The expanding and the balanced growth modes observed in keratinocytes grown in monolayer have been shown to broadly recapitulate the growth dynamics in epithelial tissues *in vivo* [28]. During the expanding growth mode in sub-confluent cells, which is suggestive of a wound-healing environment in the epithelium [28, 52], both HPV16 and HPV11 genomes are replicated and maintained in a E6/E7- and E1-independent manner. In contrast, when a balanced growth mode was triggered, which resembles the differentiation dynamics regulating stratified epithelial homeostasis [28, 53]), the HPV replication mode switched towards E1-dependency and required the functions of the accessory genes, E6 and E7. It is reasonable to speculate that a balanced growth mode is followed in at least a proportion of basal keratinocytes, since the commitment to differentiation has been shown to occur already in the basal layer [20, 54]. This suggests that an E1-independent replication mode might be used by HPVs to promote the long-term genome maintenance in the basal layer of the epithelium during productive infection, as well during subclinical or latent infections that may be subject to immune control. These are important considerations when evaluating possible targets for therapy, with E1 inhibitors having a role primarily as viral genome amplification occurs during epithelial differentiation, rather than ubiquitously at all stages of the virus life cycle. Interestingly, a well-studied two-phase replication strategy is also used by Epstein-Barr virus (EBV) during its latent and lytic phases. In this case, the EBV genome is replicated once per S phase in the latent phase of infection [55, 56], and at this point EBV expresses only a few viral genes and employs the host-encoded replication licensing proteins, MCMs and ORC, for replication, a process which is facilitated by the viral protein EBNA-1 [57]. Interestingly HPV E2 and EBNA-1 shares structural and functional similarities, even though the sequence are not conserved [58–61]. Recently, the interaction between HPV31 E2 and ORC2 has been reported, although its contribution to viral genome replication is unclear [62]. Our data indicate that HPV may also have a latent replication phase, in which the viral genome is replicated once per S phase mediated by E2 (Fig 4). It is currently unknown whether and how MCMs and ORCs are involved in HPV DNA replication, but we speculate that the E1-independent/E2-dependent maintenance replication employs the cellular replication machinery to support viral genome maintenance under S phase control. In addition, E1- and E2-independent cis-replicating elements may also reside outside the LCR and possibly in the late region (L2-L1 open reading frames [ORFs]) of the HPV16 genome [63].

Our studies also suggest functional similarities in the way that the HPV E6 and E7 proteins contribute to the phenotype of the infected basal cell, by modifying the cellular environment to facilitate genome replication. E6 and E7 impact on a range of biological events, including cell survival, transcription/translation, host cell differentiation, growth factor dependence, DNA damage responses, and cell cycle progression [11, 12]. In this study, we directly compared the early stages of the high- and low-risk HPV life cycles in a common keratinocyte background. It is clear from this that E6 plays a critical role in early differentiating keratinocytes, as the HPV16 E6SAT mutant genome, which has intact E7 but is incompetent for p53 degradation, failed to support E1-dependent replication in differentiating cells. E7 undoubtably has an important role during the later stages of differentiation, although this was not modelled as part of this study. Our data also link HPV genome replication to the inhibition of Notch signalling [20]. The E6 proteins of both high and low-risk alpha papillomaviruses have evolved to stimulate the degradation of p53 (Fig 6A), albeit with different mechanisms (especially in E6AP-dependency), dynamics and effects expected on Notch signalling (see also [20, 37]). Interestingly, E6 proteins expressed by cutaneous papillomaviruses belonging to the Beta, Delta and Mu genera associate with MAML1, a Notch transcriptional co-activator, in order to repress Notch signal transduction [64–66]. Indeed, we suspect that use of these different regulatory pathways may reflect the different roles of p53 in regulating differentiation according to epithelial sites, and the adaptation of HPVs to different epithelial niches where the role of p53 is more or less important. In fact, p53 levels in the basal and parabasal layers of normal ectocervical epithelium are high when compared to normal dermal epithelium [20], which suggests that this epithelial site is in fact normally regulated by p53, and that p53 may have a fundamental role in normal homeostatic control at this site [20]. E6 is also expected to play a role in countering p53-dependent growth arrest and/or apoptosis of course, especially during E1-dependent genome amplification, where DNA damage sensors ATR or ATM DDR [67, 68] are triggered. When taken together, our data suggest that HPV 11 and HPV 16, which are both Alphapapillomavirus, but which are categorized as low or high risk, make use of similar strategies for viral gene replication. During maintenance replication, which occurs in cycling cells in the absence of differentiation signals, the requirement for viral factors appears minimal, with E1-independent/E2-dependent replication occurring in the absence of a requirement for E6-mediated p53 degradation. Once cells reach confluence, they commit to differentiation as a result of cellular contact inhibition. In this environment, the HPV E6/E7 accessory genes modulate the cellular environment to facilitate E1-dependent replication by inhibiting differentiation as a result of E6-mediated p53 degradation, which is a characteristic of both the high and low-risk Alpha HPV E6 proteins. A clear role for E7 in driving cell cycle re-entry for productive genome replication is apparent across different HPV types. A role in evasion of the innate immune response and in regulating virus tropism are other important functions that we expect to be mediated primarily by E6 and E7.

## Supporting information

S1 Fig Validation of the episomal status of HPV genomes in NIKS. Representative Southern blot showing the presence of viral episomes in the NIKS cell lines. In the example shown, a HPV11 genomic probe was used to detect the HPV11WT and the HPV E1-defective genomes in NIKS and NIKS cells expressing 16E6 and/or E7, (left), and 11E6 and/or E7 (right). Tracks contain *Dpn*I / *Xho*I-digested total DNA isolated from cells collected at passage 1, 8 days after transfection. *Dpn*I and *Xho*I do not digest HPV11 genome replicated in NIKS. The *BamH*I-linearized HPV11 genome was used as copy number control (far left). OC = open circular, Ln = linear, SC = super coiled.
(TIF)

S2 Fig Expression of HPV16 and HPV11 E6 and E7 in NIKS cells. (A) NIKS or NIKS expressing 16E6 and/or 16E7 were cultured to confluence. Cell pellets were lysed using RIPA buffer, and the viral proteins detected by western blotting as described previously [24]. (B) For HPV11, E6 and E7 expression was confirmed at the RNA level following cDNA synthesis, PCR and gel electrophoresis. A reverse transcriptase (RT) negative control (no RT enzyme) was included in each case.
(TIF)

S3 Fig Requirement of E6 and E7 for genome amplification but not maintenance. (A) The virus genome copy number per cell of E6-defective HPV11 (HPV11E6def left) or HPV16 (HPV16E6def, right) transfected into NIKS was measured and prepared for presentation as outlined in Fig 1E. (B) The virus genome copy number per cell of E7-defective HPV11 (HPV11E7def left) or HPV16 (HPV16E7def, right) transfected into NIKS was measured and prepared for presentation as outlined in Fig 1E. Both the HPV11/16 E6def and HPV16 E7def genome declined post-confluence in the absence of a helicase-active E1 gene.
(TIF)

S4 Fig Change in HPV copy number per cell following passage. (A) The full genome of HPV16 WT, E1def, E6ΔPBM, HPV16E6SAT E6def or E7def was transfected into NIKS. The viral genome copy number per cell before passage (Passage 1 and 2) was set at 100%, and each relative genome copy number per cell 24 hours after passage (Passage 2 and 3) is shown. For the HPV16 genomes, only the WT genome was maintained at equivalent levels following passage of cells in tissue culture. Loss of E1 or E6 functions compromised genome maintenance when cells were subject to passage stress. (B) The virus genome copy number per cell of HPV11 transfected NIKS or NIKS expressing 16E6, 16E6E7 or 16E7 was measured as outlined in Fig 1E. The viral genome copy number per cell before passage was set at 100%, and each relative genome copy number per cell 24 hours after passage (Passage 2 and 3) was shown. The WT HPV11 genome, which declines following passage, is not rescued following expression of HPV16 E6 and/or E7, even though these genes prevent copy number loss in confluent cells.
(TIF)

S5 Fig Expression of p53 in the parental NIKS cell line. NIKS cells at days 3 and 7 were stained with p53, and with DAPI (blue) as a nuclear counterstain.
(TIF)

S6 Fig HPV11 E6 drives a reduction in p53 levels by E6AP independent manner. (A) NIKS or NIKS expressing HPV16 E6, 11E6WT, 11E6W133R, which cannot bind to E6AP are shown at the day 7 time point (post-confluence) following staining for p53 (red) and DAPI (blue). (B) NIKS expressing 11E6WT and shRNA targeted against E6AP were stained at day 7 for p53 and DAPI (upper image). The reduction of E6AP level in cells expressing the E6AP shRNA was confirmed by western blotting (lower image). (C) The proportion of P53 positive cells shown in (A) and (B) are quantified and shown as a bar chart.
(TIF)
