# Supplementary figures and images for: Roles for E1-independent replication and E6-mediated p53 degradation during low-risk and high-risk human papillomavirus genome maintenance
Source: PLoS Pathog. 2019 May 13;15(5):e1007755. doi: 10.1371/journal.ppat.1007755 (PMC6544336; doi:10.1371/journal.ppat.1007755)

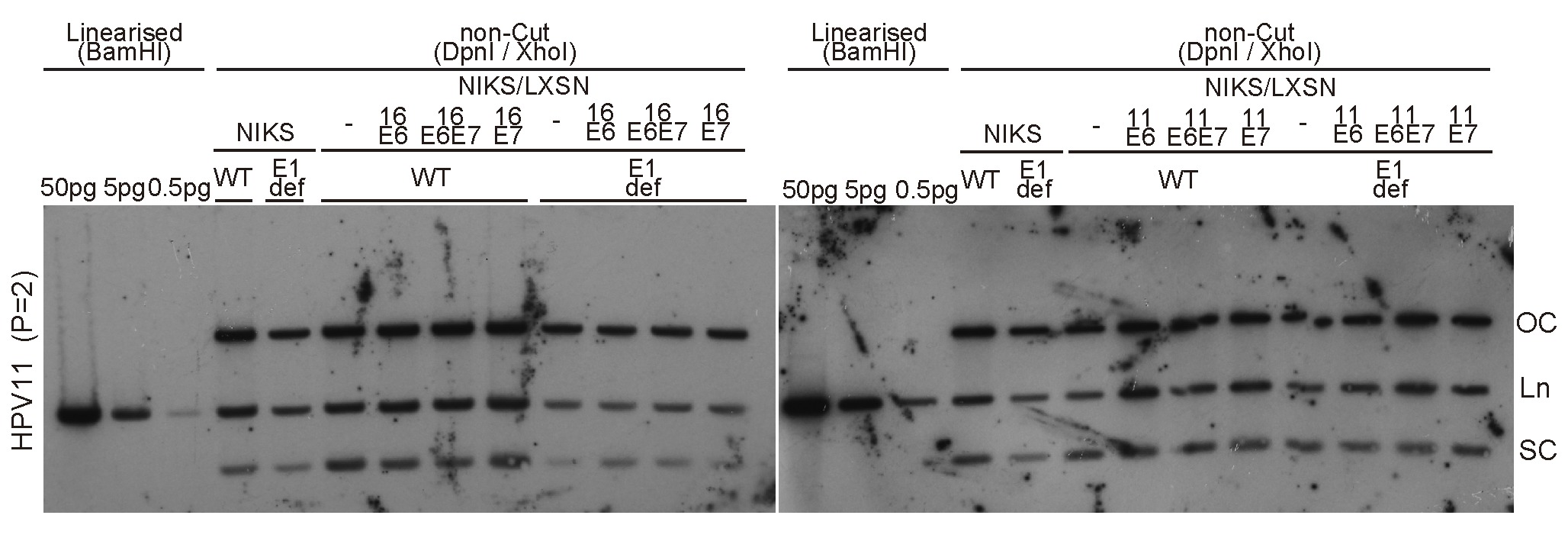

Supplement: S1 Fig — Representative Southern blot showing the presence of viral episomes in the NIKS cell lines. In the example shown, a HPV11 genomic probe was used to detect the HPV11WT and the HPV E1-defective genomes in NIKS and NIKS cells expressing 16E6 and/or E7, (left), and 11E6 and/or E7 (right). Tracks contain DpnI / XhoI-digested total DNA isolated from cells collected at passage 1, 8 days after transfection. DpnI and XhoI do not digest HPV11 genome replicated in NIKS. The BamHI-linearized HPV11 genome was used as copy number control (far left). OC = open circular, Ln = linear, SC = super coiled. (TIF) [file ppat.1007755.s001.tif]

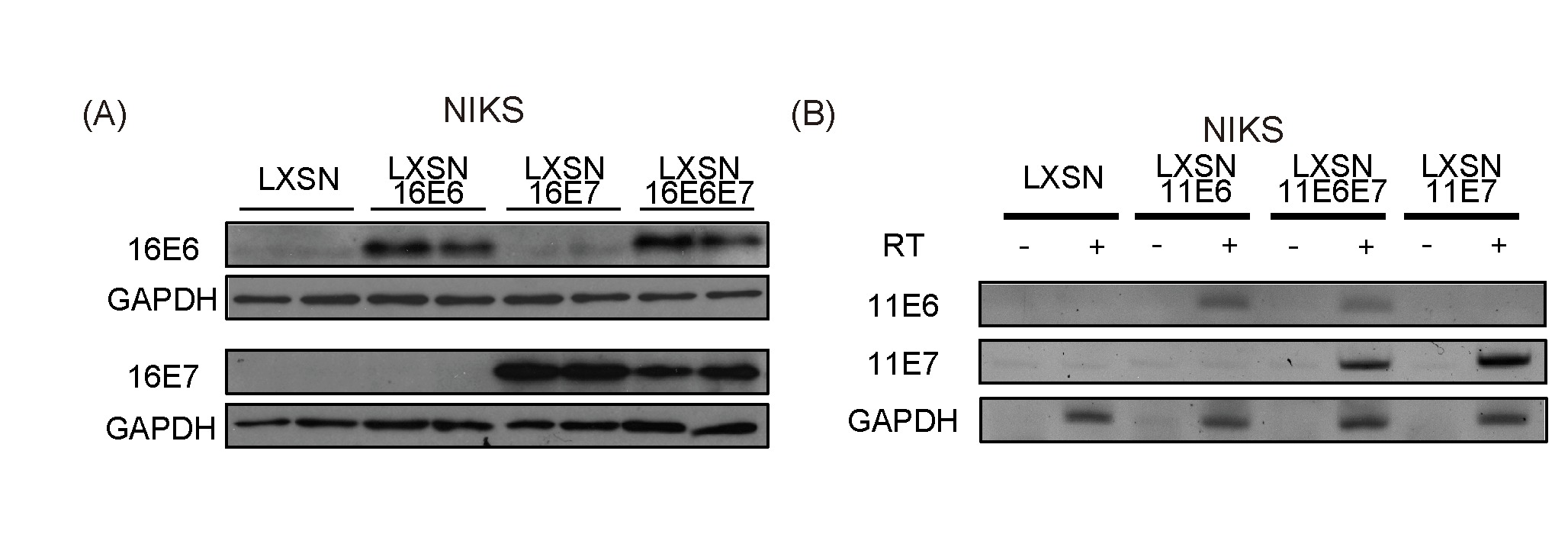

Supplement: S2 Fig — (A) NIKS or NIKS expressing 16E6 and/or 16E7 were cultured to confluence. Cell pellets were lysed using RIPA buffer, and the viral proteins detected by western blotting as described previously [24]. (B) For HPV11, E6 and E7 expression was confirmed at the RNA level following cDNA synthesis, PCR and gel electrophoresis. A reverse transcriptase (RT) negative control (no RT enzyme) was included in each case. (TIF) [file ppat.1007755.s002.tif]

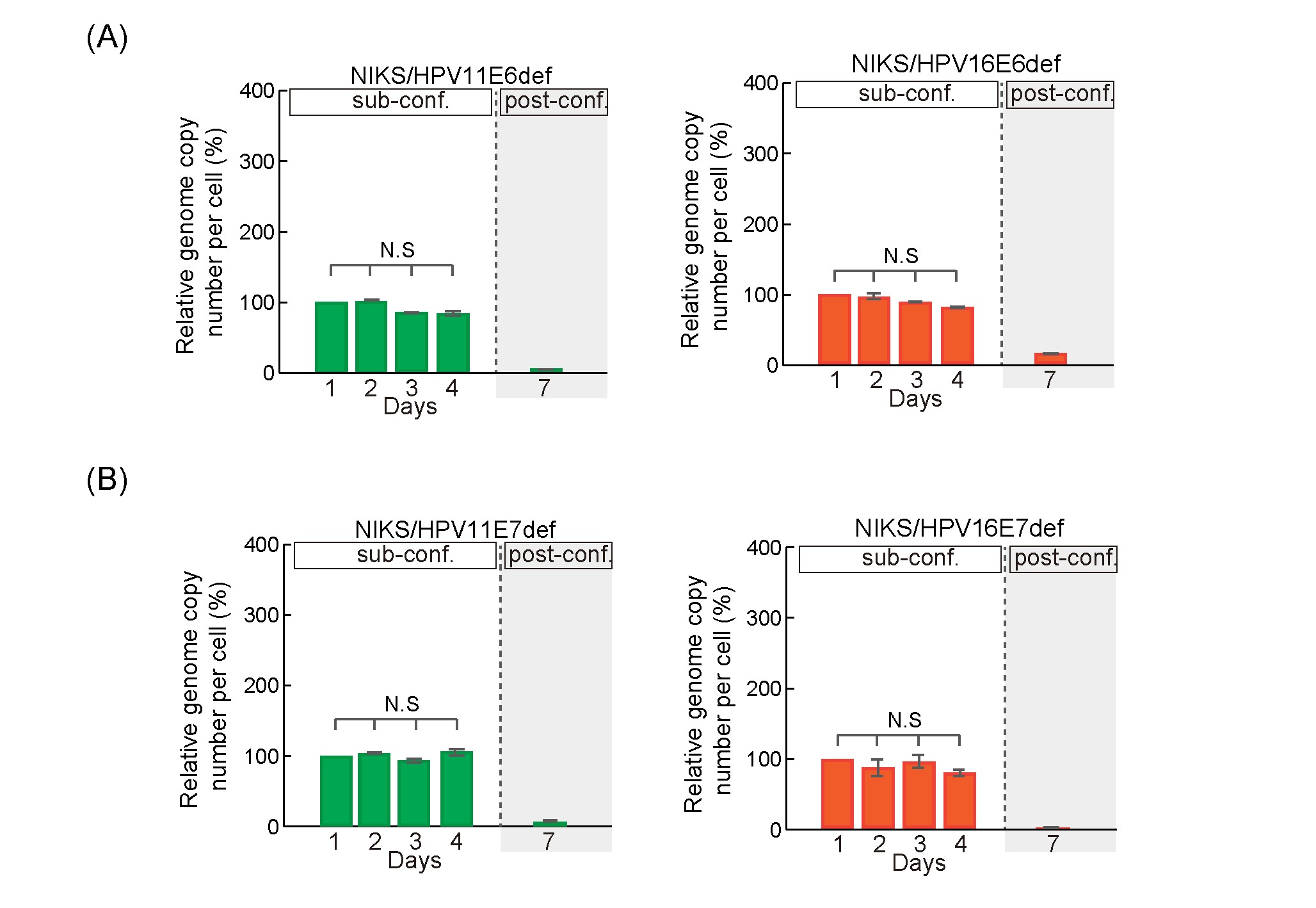

Supplement: S3 Fig — (A) The virus genome copy number per cell of E6-defective HPV11 (HPV11E6def left) or HPV16 (HPV16E6def, right) transfected into NIKS was measured and prepared for presentation as outlined in Fig 1E. (B) The virus genome copy number per cell of E7-defective HPV11 (HPV11E7def left) or HPV16 (HPV16E7def, right) transfected into NIKS was measured and prepared for presentation as outlined in Fig 1E. Both the HPV11/16 E6def and HPV16 E7def genome declined post-confluence in the absence of a helicase-active E1 gene. (TIF) [file ppat.1007755.s003.tif]

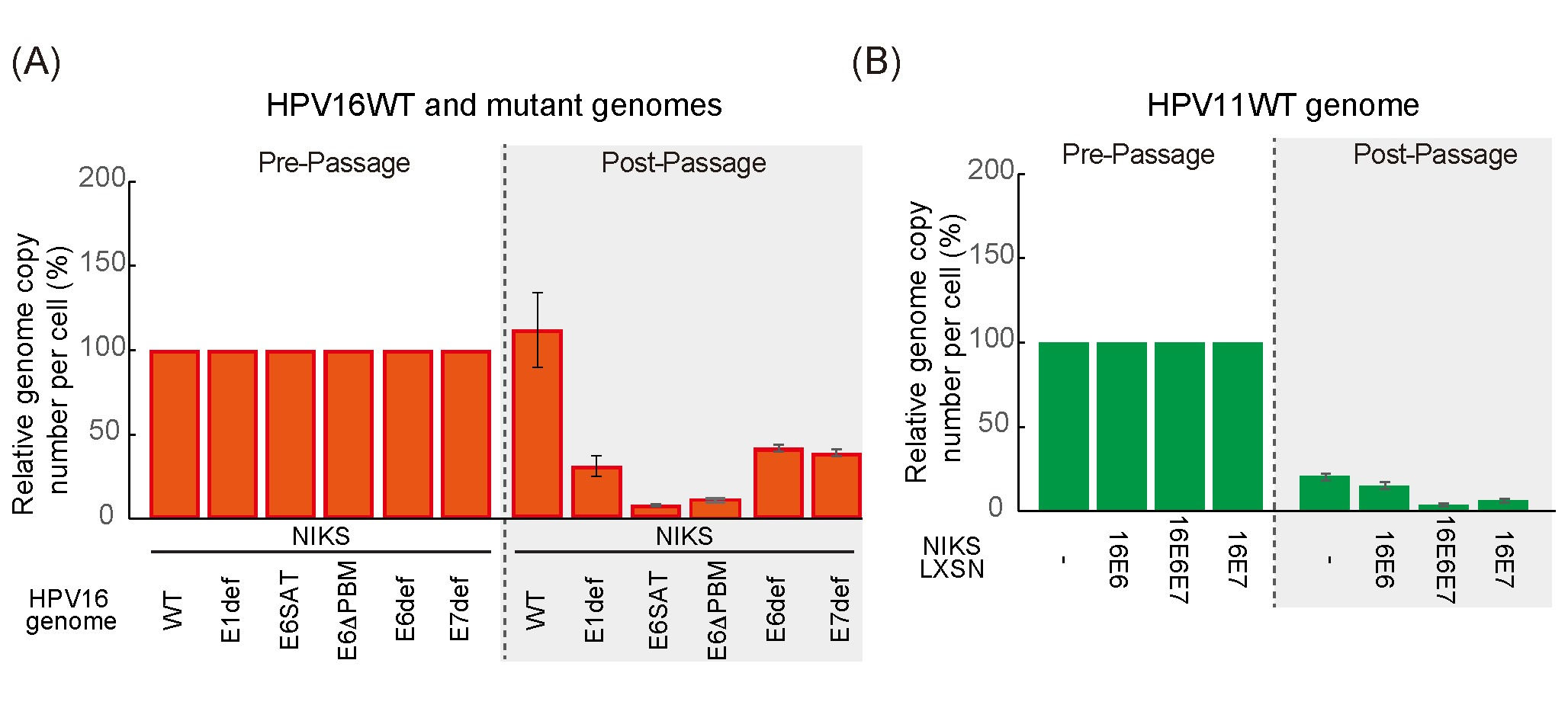

Supplement: S4 Fig — (A) The full genome of HPV16 WT, E1def, E6ΔPBM, HPV16E6SAT E6def or E7def was transfected into NIKS. The viral genome copy number per cell before passage (Passage 1 and 2) was set at 100%, and each relative genome copy number per cell 24 hours after passage (Passage 2 and 3) is shown. For the HPV16 genomes, only the WT genome was maintained at equivalent levels following passage of cells in tissue culture. Loss of E1 or E6 functions compromised genome maintenance when cells were subject to passage stress. (B) The virus genome copy number per cell of HPV11 transfected NIKS or NIKS expressing 16E6, 16E6E7 or 16E7 was measured as outlined in Fig 1E. The viral genome copy number per cell before passage was set at 100%, and each relative genome copy number per cell 24 hours after passage (Passage 2 and 3) was shown. The WT HPV11 genome, which declines following passage, is not rescued following expression of HPV16 E6 and/or E7, even though these genes prevent copy number loss in confluent cells. (TIF) [file ppat.1007755.s004.tif]

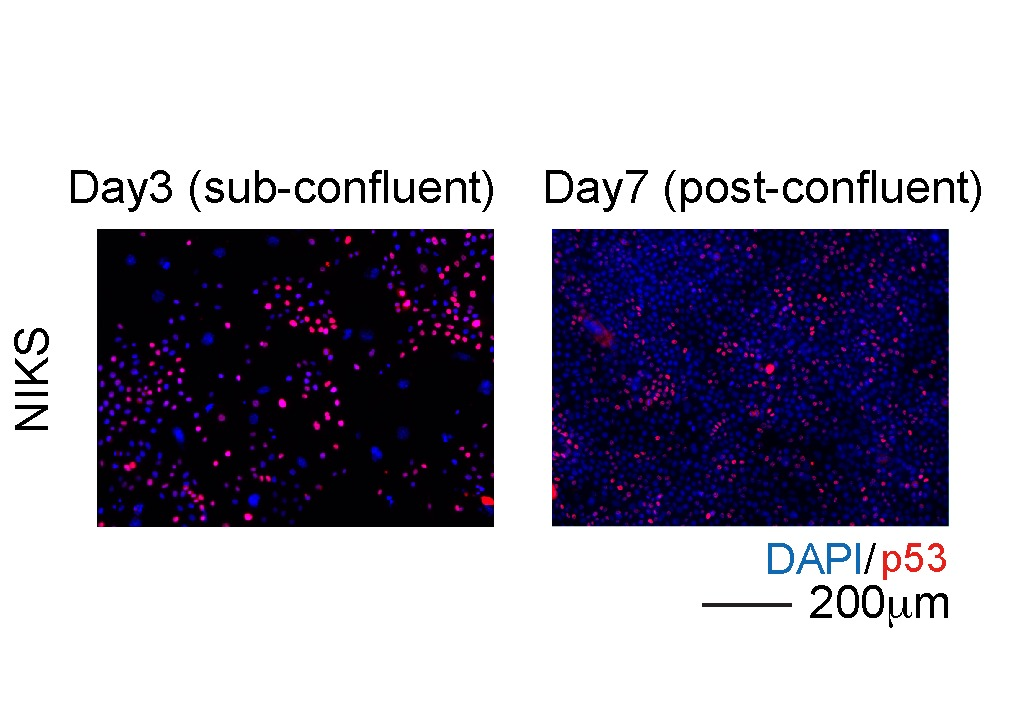

Supplement: S5 Fig — NIKS cells at days 3 and 7 were stained with p53, and with DAPI (blue) as a nuclear counterstain. (TIF) [file ppat.1007755.s005.tif]

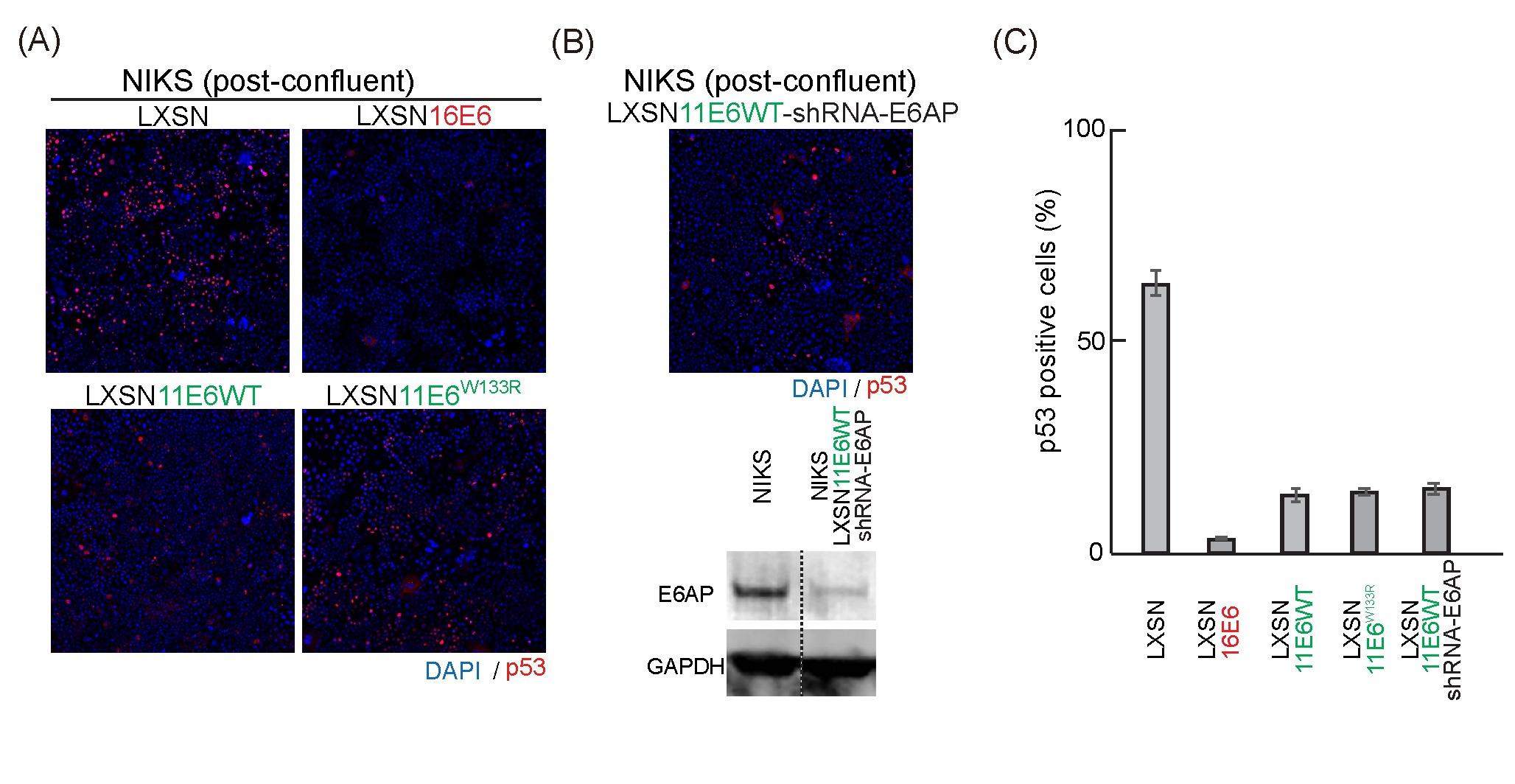

Supplement: S6 Fig — (A) NIKS or NIKS expressing HPV16 E6, 11E6WT, 11E6W133R, which cannot bind to E6AP are shown at the day 7 time point (post-confluence) following staining for p53 (red) and DAPI (blue). (B) NIKS expressing 11E6WT and shRNA targeted against E6AP were stained at day 7 for p53 and DAPI (upper image). The reduction of E6AP level in cells expressing the E6AP shRNA was confirmed by western blotting (lower image). (C) The proportion of P53 positive cells shown in (A) and (B) are quantified and shown as a bar chart. (TIF) [file ppat.1007755.s006.tif]
